# Computer aided chemical design: using quantum chemical calculations to predict properties of a series of halochromic guaiazulene derivatives

**DOI:** 10.1098/rsos.160373

**Published:** 2016-11-23

**Authors:** Adam W. Woodward, Ebrahim H. Ghazvini Zadeh, Mykhailo V. Bondar, Kevin D. Belfield

**Affiliations:** 1 Department of Chemistry, University of Central Florida, Orlando, FL 32816-2366, USA; 2 Institute of Physics NASU, Prospect Nauki, 46, Kiev 03028, Ukraine; 3 College of Science and Liberal Arts, New Jersey Institute of Technology, University Heights, Newark, NJ 07102, USA; 4 School of Chemistry and Chemical Engineering, Shaanxi Normal University, Xi'an 710062, People's Republic of China

**Keywords:** halochromic, guaiazulene, two-photon absorption

## Abstract

With the scientific community becoming increasingly aware of the need for greener products and methodologies, the optimization of synthetic design is of greater importance. Building on experimental data collected from a synthesized guaiazulene derivative, a series of analogous structures were investigated with time-dependent density functional theory (TD-DFT) methods in an effort to identify a compound with desirable photophysical properties. This *in silico* analysis may eliminate the need to synthesize numerous materials that, when investigated, do not possess viable characteristics. The synthesis of several computationally investigated structures revealed discrepancies in the calculation results. Further refined computational study of the molecules yielded results closer to those observed experimentally and helps set the stage for computationally guided design of organic photonic materials. Three novel derivatives were synthesized from guaiazulene, a naturally occurring chromophore, exhibiting distinct halochromic behaviour, which may have potential in a switchable optoelectronic system or combined with a photoacid generator for data storage. The protonated forms were readily excitable via two-photon absorption.

## 1. Introduction

Building on conventional chemistry, effective solutions for greener chemical practices are increasingly being implemented to tackle concerns of chemical hazards, resource scarcity and climate impact on the population and the planet. In his 1998 book, Paul Anastas features 12 principles for green chemistry, the first of which states: ‘It is better to prevent waste than to treat or clean waste after it is formed’ [1]. To this end, the use of quantum chemical calculations to provide a prediction of a compound's properties prior to generating it in the laboratory could help to minimize waste generated by synthesis of impractical derivatives. The use of *in silico* techniques is commonly used to aid in the explanation of experimental results [2,3], though more recently studies have been directed towards identifying sustainable solvents [4], as well as designing solar cell components [5] and complex metal oxides [6].

Another of Anastas's principles, and area of keen development, is the use of chemicals from renewable sources, or otherwise coming from nature [1]. Extractable from fungi and coral [7,8], 1,4-dimethyl-7-isopropylazulene, or guaiazulene, is a natural derivative of azulene. Although azulene is an isomer of the colourless naphthalene, it exhibits a blue colour that has enchanted man since the late medieval period [9]. This is attributed to azulene's peculiar emission from the second excited state (S_2_), an exception to Kasha's Rule, as a result of its unusually low-lying first excited state (S_1_). Introducing electronic perturbing substituents on the seven-member ring and/or on the five-member ring of azulene was shown to change the electronic properties of azulene derivatives, accompanied by significant changes in their fluorescence behaviour [9,10]. Although the effect of resonantly electron withdrawing or donating groups on the HOMO, LUMO and LUMO +1 energies of azulene was reported, these theoretical calculations were initially limited to derivatives with mildly electronically perturbing (e.g. formyl or fluorine) substituents [11].

**Figure 1.**
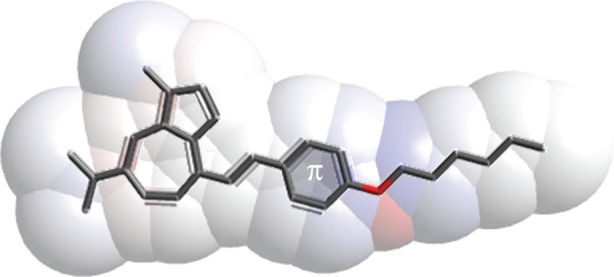
Generic structure of a guaiazulene-terminated compound with a varied π-bridge.

Recently, a number of studies have reported interesting optoelectronic properties of azulene derivatives having extended π-conjugated substituents that can be manipulated by protonation with strong acids. The formation of a resonance-stabilized 6-π-electron tropylium cation in protonated azulenes [12] resulted in a bathochromic shift in the S_0_ → S_1_ band of the absorption, as well as an increase in the luminescence intensity as a result of the now-dominant S_1_ → S_0_ decay pathway [13,14]. The unique electronic properties of azulene-based structures were instrumental in the development of derivatives for charge-transport, optoelectronic and sensor applications. The design of such structures is often based on theoretical calculations of the dipole moment of azulene derivatives; variation in calculated dipole moments is thought to be a result of various substituents on the azulene framework that acts as an electron bridge in a donor–acceptor–donor arrangement [12,15,16]. While these calculations predict a large dipole moment and large hyperpolarizability for 2,6-connected azulene systems, experimental investigation shows that a 4,7-connectivity often results in longer absorption and emission wavelengths, a desirable property for the development of various near-IR applications [10]. In order to avoid such discrepancies and minimize waste generated by preparing potentially impractical azulene derivatives, we exploited the naturally occurring guaiazulene to initially prepare a structurally simplified derivative tailored to guide the theoretical calculations to predict critical optical properties of more heavily conjugated systems (figure 1). Such *in silico* analysis could reveal any disadvantageous nature of certain derivatives, thus eliminating the wasteful necessity of producing derivatives with undesirable properties.

**Figure 2.**
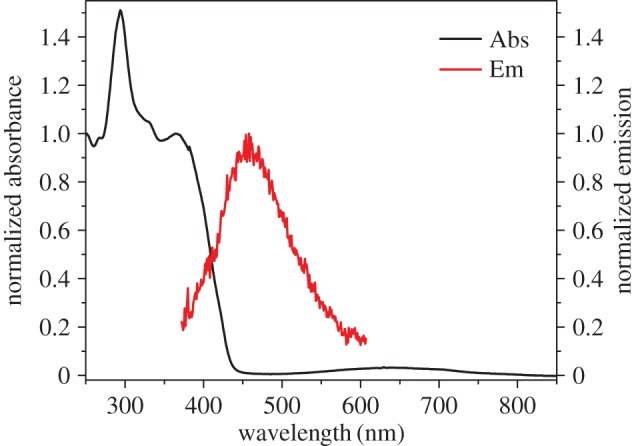
Absorption (black) and emission (red) spectra of **3a** in dichloromethane (DCM).

**Figure 3.**
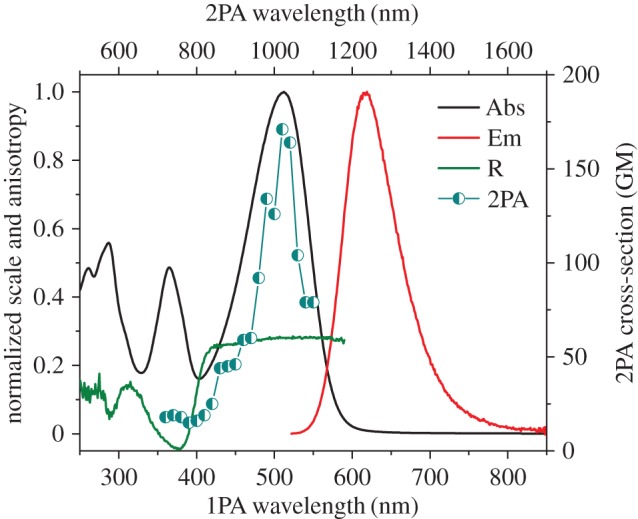
Absorption (black), emission (red), and 2PA spectra (blue circles) for **3aH+** in 10% TFA/DCM, and excitation anisotropy (green) in acidified silicone oil.

**Scheme 1.**
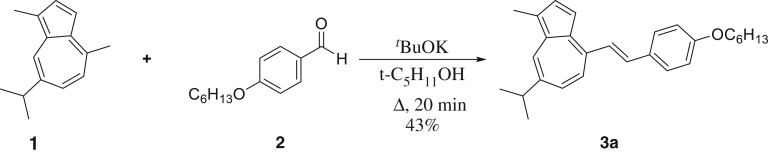
Synthesis of guaiazulene **3a**.

**Scheme 2.**
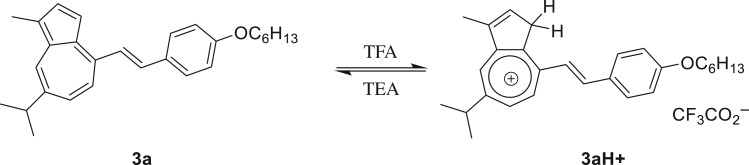
Structures for guaiazulene derivative **3** and its conjugate acid formed upon exposure to TFA, **3aH+**.

In its protonated state, azulene's tropylium cation acts as an electron acceptor when conjugated through a π-bridge to an electron-rich system. With that in mind, and using our established method [17], guaiazulene **1** was condensed with 4-hexyloxybenzaldehyde **2a** in the presence of potassium tert-butoxide to afford **3a** (scheme 1). Thus, upon treatment with trifluoroacetic acid (TFA), the ethylene moiety acts as a π-spacer between the tropylium cation and the electron-rich benzene ring (scheme 2). This is illustrated in the absorption and emission spectra of **3a** and its protonated form **3aH+** (figures 2 and 3). In addition to the weak S_0_ → S_1_ transition (depicted as a broad peak centred at 630 nm) common to the azulene family, **3a** shows an absorption peak with λ_max_ = 365 nm, hence giving the solution of **3a** a typical azure blue colour. On the other hand, an intense red colour is immediately observed when the solution is treated with TFA. This is reflected in the absorption spectrum of **3aH+** where an intense peak appears at λ_max_ = 512 nm, while that of the neutral species **3a** (λ_abs_ = 630 nm) is no longer observed. Concomitantly, treatment of **3a** with TFA resulted in a marked switch-on of fluorescence; in fact, a quantum yield of 0.12 was calculated for **3aH+** using the Lorentz–Lorenz equation (1.1) and (1.2), where OD is optical density, *I* is emission intensity, *n_x_* is the refractive index of solvent *x*, and *φ_x_* is the proportion of solvent *x* in the mixture [18,19]. As seen in figure 3, a prominent emission peak is seen at λ_max_ = 620 nm, which was chosen in order to measure the excitation anisotropy for the protonated species **3aH+** in a viscous medium (figure 3). The plateau of this trace through the main absorption band indicates that this is a single electronic transition (figure 3). Additionally, it was noted that acid treatment also results in **3a** having significant two-photon absorptivity, with a two-photon absorption cross-section of approximately 170 GM at 1020 nm (figure 3). Given the unsymmetrical design of the molecule, the two-photon absorption (2PA) spectrum agrees well with that of the linear absorption.

1.1 $$\begin{array}{rl} \Phi_{f} & =\Phi_{\mathrm{ref}}\frac{OD_{\mathrm{ref}}}{\mathrm{OD}}\frac{I}{I_{\mathrm{ref}}}\frac{n^{2}}{n_{\mathrm{ref}}^{2}} \end{array}$$

1.2 $$\begin{array}{rl} \frac{n_{1,2}^{2}-1}{n_{1,2}^{2}+2} & =\phi_{1}\frac{n_{1}^{2}-1}{n_{1}^{2}+2}+\phi_{2}\frac{n_{2}^{2}-1}{n_{2}^{2}+2} \end{array}$$

The quantum yields of photodecomposition measured for **3a** and **3aH+** show significant disparity, separated by three orders of magnitude (table 1). Though a value is not explicitly stated, the photostability of guaiazulene has been investigated previously; the sample was seen to persist for a longer period of time than **1** at an equivalent or greater irradiance but no quantitative value was reported [20]. It is possible that change of the solvent from methanol to dichloromethane (DCM) is responsible for the discrepancy: DCM can promote photooxidation and C–Cl bonds can be broken using UV light, leading to reactive species and additional damage to the compound in solution [21]. In the case of **3aH+**, longer wavelength light was used, thus avoiding the degradation of the solvent, and obtaining a value similar to those of other fluorescent dyes that have been previously reported [22].

**Table 1.** Photophysical parameters measured for **3a** and **3aH+** in DCM and 10% TFA/DCM, respectively.

	$\lambda_{\max}^{\mathrm{abs}}$^a^ (nm)	$\lambda_{\max}^{\mathrm{em}}$^a^ (nm)	*Δλ*^b^ (nm)	ε^c^ (M^−1^ cm^−1^)	*Φ*^d^_f_	*τ*^e^ (ns)	*Φ*_Ph_ (10^−6^)
**3a**	365, 630	458	93	29 000, 690	<0.01	—^f^	1000
**3aH+**	512	619	93	25 000	0.12	0.87	1.5

^a^Absorption and emission maxima ±1 nm.

^b^Stokes shift ±2 nm.

^c^Extinction coefficients ±5%.

^d^Fluorescence quantum yields ±10%.

^e^Fluorescence lifetimes ±10%.

^f^Not determined.

**Figure 4.**
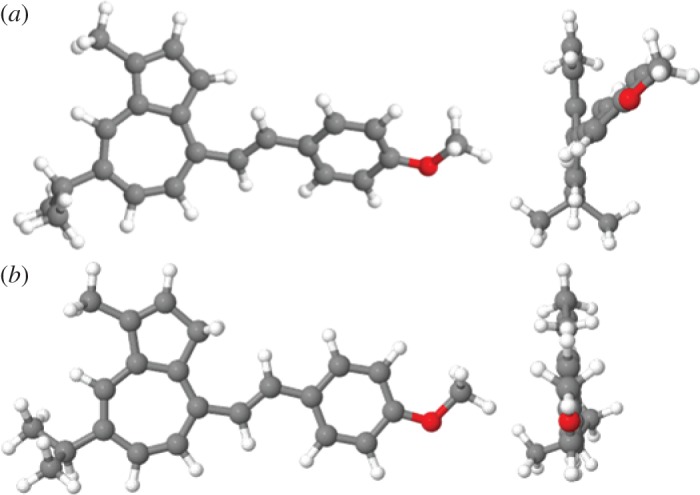
Optimized structures for **3a** (*a*) and **3aH+** (*b*).

Examination of the optimized structures for **3a** and **3aH+** reveals that the neutral form does not exhibit a planar conformation (figure 4*a*), but, instead, a distinct dihedral angle, *ϕ*, between the guaiazulene moiety and the vinyl bond (*ϕ *≈* *38°) is noted. When guaiazulene is protonated, the optimized structure indicates that planarity is restored (*ϕ *≈* *1°, figure 4*b*), hence amplifying electron donation from the adjacent anethole group.

The ground state stationary dipole for the two forms is consistent in magnitude (1.90 D and 2.20 D for **3a** and **3aH+**, respectively) despite the presence of an electron deficient tropylium cation in **3aH+**. However, the dipole of **3aH+** is polarized toward the cyclopentene ring rather than the anethole moiety.

**Figure 5.**
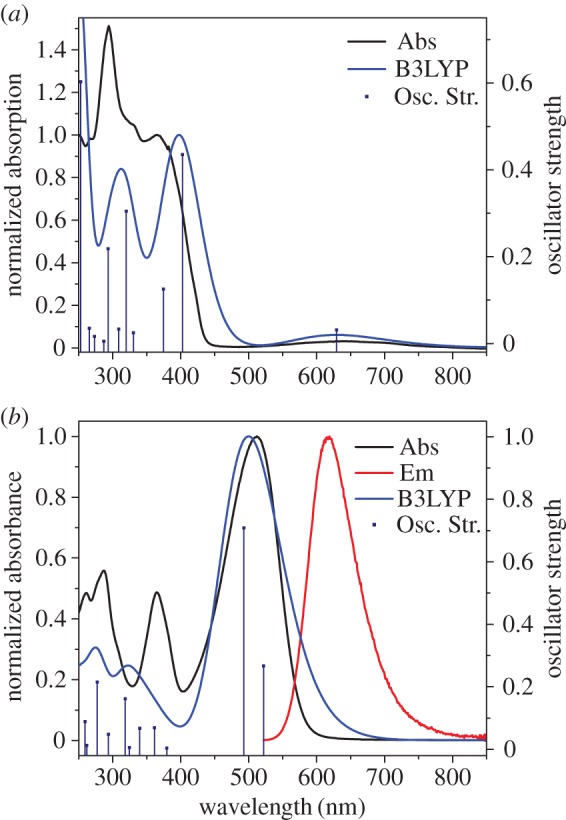
Calculated absorption spectra (blue line) and oscillator strengths (blue bars) overlaid with experimental absorption (black) and emission (red) spectra for **3a** (*a*) and **3aH+** (*b*).

The absorption spectra generated through time-dependent density functional theory (TD-DFT) calculations show that the weak intensity band of the S_0_ → S_1_ transition is present, though the bands at shorter wavelength are resolved better than seen experimentally (figure 5*a*; electronic supplementary material, table S1). In the case of **3aH+** (figure 5*b*; electronic supplementary material, table S2), the absorption band centred at approximately 600 nm is anticipated by the calculation; however, the short wavelength bands are less well aligned.

**Figure 6.**
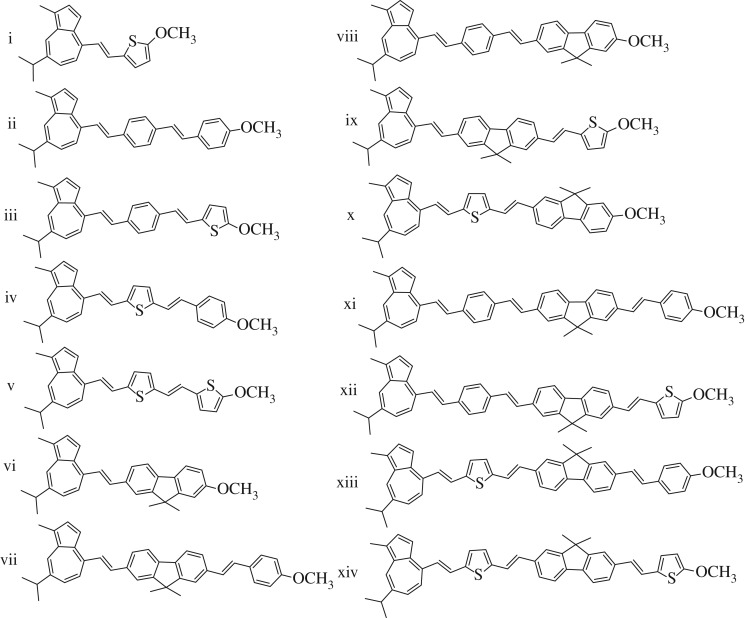
Compounds designed using **3** as a basis.

The consistency found between the experimental and calculated photophysical properties of **3a** could, therefore, allow for prediction of the electronic properties of a series of structures similar to **3a** and having various π-spacers (figure 6). Among potential spacers, phenyl rings offer a greater degree of rigidity over polyene groups; compared with these spacers, the fluorene moiety can be a better alternative as it confers higher rigidity given the bridging carbon in the 9-position. Alternatively, the use of thiophene, an isostere of benzene, is shown to result in a bathochromic shift of the linear absorption spectrum [21,22]. The length of conjugation is known to shift the absorption spectrum, but it can also lead to increased two-photon absorption cross-sections [23,24]. Eleven structures were studied in their neutral and protonated forms. Of this π-extended series, two structures were synthesized and their experimentally determined properties were compared with calculated properties.

Analysis of the results from TD-DFT calculations (table 2) reveals that the varying length of the chromophore system has little effect on the ground state dipole of the neutral form for this series. However, the protonated forms of the longer systems (vi–xiv) show a marked increase relative to their neutral counterparts, ranging from approximately threefold to eightfold increases. There is also a notable trend in the dihedral angles observed for the optimized structures: the structures with a thiophene ring adjacent to the guaiazulene system (i, iv, v, x and xiii) do not exhibit a change in dihedral angle between these systems upon protonation, instead both forms are planar. Conversely, structures with either a phenyl ring or fluorene moiety adjacent to the guaiazulene undergo approximately 25° rotation to relax the dihedral angle upon protonation. A possible explanation is that thiophene withdraws electron density from the vinyl hydrogen closest to the guaiazulene, thereby facilitating a planar conformation of the neutral form. Structure xiv, however, appears to be an exception to this trend, behaving similarly to the phenyl-substituted guaiazulenes.

**Table 2.** TD-DFT calculation data for the series of compounds shown in figure 6.

Cmpd	dipole (D)	dihedral angle	first transition (nm)	$\lambda_{\max}^{\mathrm{abs}}$ (nm)
*neutral forms*				
i	1.62	0.639	639	418
ii	3.13	23.0	650	459
iii	3.11	19.5	653	485
iv	3.14	0.074	677	493
v	4.32	0.001	679	510
vi	2.45	22.7	637	437
vii	3.68	24.1	644	476
viii	3.02	18.8	657	487
ix	2.59	37.0	647	405
x	3.59	0.553	682	519
xi	3.11	17.2	661	508
xii	2.00	23.1	656	448
xiii	3.15	0.050	686	540
xiv	1.39	17.8	685	557
*protonated forms*				
iH+	1.39	0.024	527	483
iiH+	8.34	5.05	753	738
iiiH+	6.56	3.56	758	732
ivH+	6.13	0.040	683	668
vH+	4.24	0.017	688	668
viH+	8.48	0.004	676	657
viiH+	15.6	0.000	1009	999
viiiH+	16.7	0.016	966	949
ixH+	13.0	0.011	1048	1041
xH+	13.9	0.011	847	842
xiH+	26.1	0.012	1473	730
xiiH+	23.9	0.001	1563	736
xiiiH+	23.0	0.004	1273	688
xivH+	19.1	0.006	1354	709

**Figure 7.**
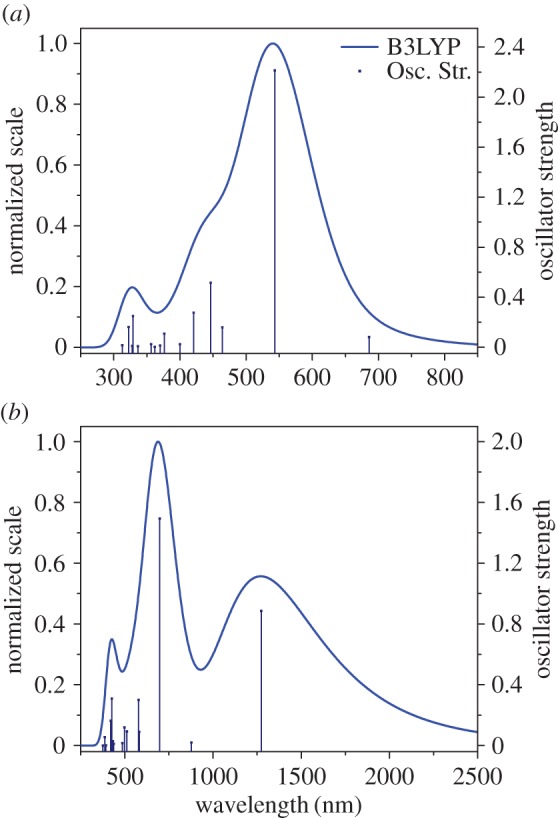
Calculated absorption spectra for xiii (*a*) and xiiiH+ (*b*) using the B3LYP method.

These results also include the first transition wavelength and the peak of the calculated absorption spectrum. Although there is some fine variation, the first transition of the neutral forms, corresponding to the S_0_ → S_1_ transition, remains in the region of 650–680 nm, consistent with experimental results for **3a** and other studies of azulene derivatives [9,12,14]. Results generally indicate that the absolute λ_max_ steadily increased as the length of the chromophore increased, as expected (figure 7*a*). This implies that the energy gap between S_0_ and S_2_ is decreasing while that between S_0_ and S_1_ remains constant.

Protonation induces the same bathochromic shift seen for compound **3a** in the calculated absorption data. Disappearance of the weak S_0_ → S_1_ band of the azulene at approximately 650 nm and emergence of a new band from the azulenium cation extending into the NIR are observed [23]. In the case of the longer species, such as **xiiiH**, the first transition no longer represents the peak absorption as it has been superseded by a higher transition (figure 7*b*).

To confirm the validity of these results, as well as probe further into the photophysical properties of these extended guaiazulene derivatives, compounds similar to structures **vii** and **xiii** were synthesized and characterized. These compounds were selected as they represent the midpoint and the extreme of the series, as well as having two and three moieties, respectively, in addition to guaiazulene. Given the increased length of the conjugated systems, and the inclusion of a thiophene moiety (in the case of **xiii**), these structures should have greater 2PA properties over **3a**.

**Scheme 3.**
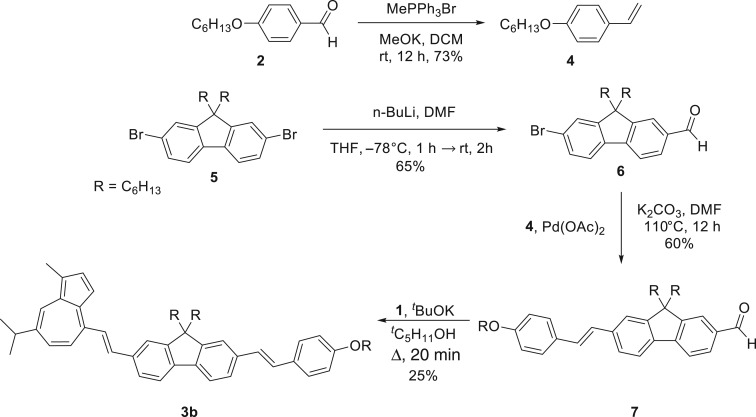
Synthetic scheme for compound **3b**.

The approach for the synthesis of **3b** is depicted in scheme 3. In this synthetic route, a Wittig reaction was employed to prepare styrene **4** by condensing benzaldehyde **2** with methyltriphenylphosphonium bromide under basic conditions. Alternatively **6**, which was prepared by a lithium-halogen exchange reaction employing intermediate **5** in the presence of *n*-BuLi, was coupled with **4** to furnish the aldehyde **7**. Finally, a condensation of **7** with guaiazulene **1** afforded the target compound **3b**. It is noteworthy that the formation of **3b** can be preliminarily observed as change in colour of the spot corresponding to the desired product from green to dark blue when exposing the compound spotted on a thin layer chromatography (TLC) plate to TFA vapour.

**Scheme 4.**
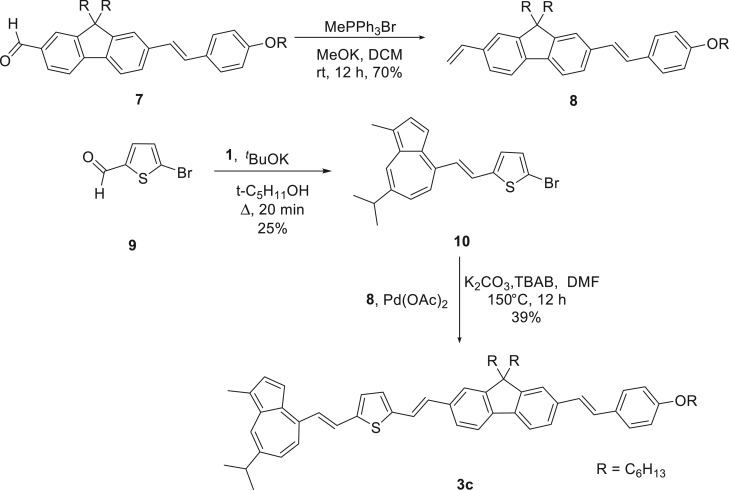
Synthetic scheme for compound **3c**.

Similarly, the synthesis of guaiazulene **3c** involved a convergent approach (scheme 4). Namely, intermediate **7** was transformed into the corresponding vinyl derivative **8**; commercially available 5-bromo-thiophene-2-carboxyaldehyde **9** was condensed with guaiazulene **1** to afford **10** bearing a bromo substituent. The two components **8** and **10** were then subjected to Heck cross-coupling conditions to furnish π-extended guaiazulene **3c**.

**Figure 8.**
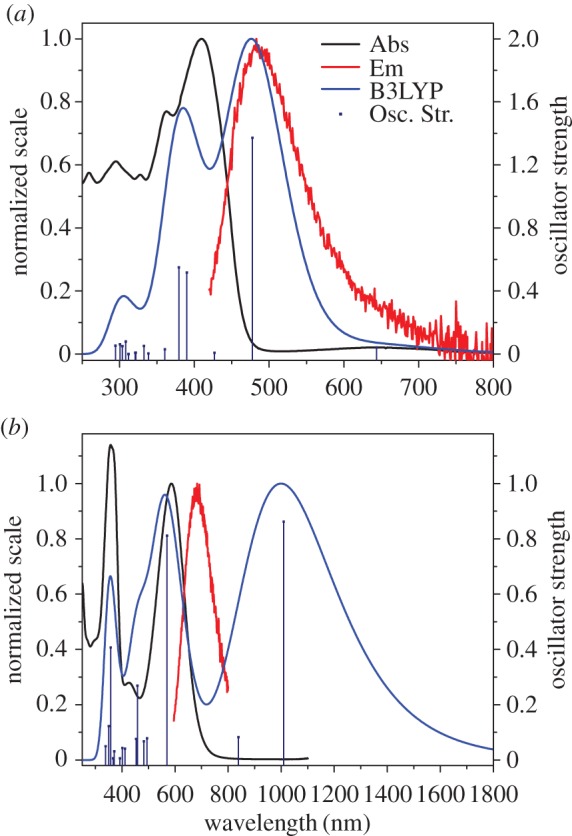
Absorption (black) and emission (red) spectra recorded for **3b** in DCM (*a*) and **3bH+** in 10% TFA/DCM (*b*) overlaid with their respective calculated absorption spectra (blue line) and oscillator strengths (blue bars).

**Figure 9.**
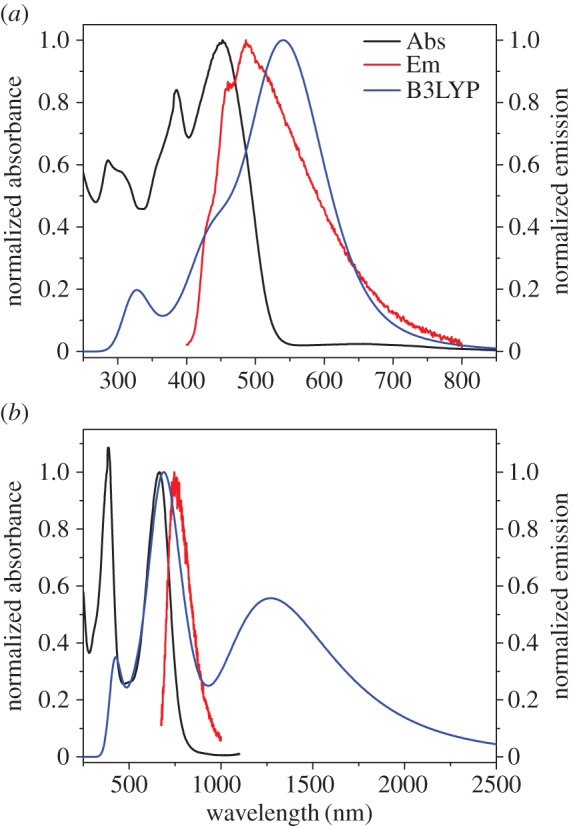
Absorption (black) and emission (red) spectra recorded for **3c** in DCM (*a*) and **3cH+** in 10% TFA/DCM (*b*) overlaid with their respective calculated absorption spectra (blue).

The experimental absorption spectra of **3b** and **3c** appear hypsochromically shifted in relation to their computationally determined counterparts (electronic supplementary material, tables S3–S6), though they share a somewhat similar structure (figures 8*a* and 9*a*). With the intense S_0_ → S_2_ bands pushed to lower wavelength, the S_0_ → S_1_ bands are more visible, appearing at approximately 650–660 nm in both instances. Weak emission was observed through excitation of the S_0_ → S_2_ band, though no emission was apparent for the S_0_ → S_1_ transition. Upon protonation, the absorption spectrum was altered in a manner similar to **3a**, though there was no evidence of the absorption bands in the NIR (figures 8*b* and 9*b*). These protonated forms also fluoresce upon excitation of the azulenium cation bands at 586 nm and 664 nm, respectively (table 3).

**Table 3.** Photophysical parameters measured for **3b** and **3c** and **3bH+** and **3bH+** in DCM and 10% TFA/DCM, respectively.

	$\lambda_{\max}^{\mathrm{abs}}$^a^ (nm)	$\lambda_{\max}^{\mathrm{em}}$^a^ (nm)	Δλ^b^ (nm)	ε^c^ (M^−1^ cm^−1^)	*Φ*_f_^d^	*τ*^e^ (ns)	*Φ*_Ph_ (10^−6^)
**3b**	408, 644	500	92	31 800, 820	0.01	—^f^	85
**3bH+**	355, 586	700	114	38 000, 25 400	0.03	2.06	—^f^
**3c**	448, 656	486	38	16 100, 470	0.10	—^f^	780
**3cH+**	385, 664	764	100	18 200, 16 900	0.01	1.52	13

^a^Absorption and emission maxima ±1 nm.

^b^Stokes shift ±2 nm.

^c^Extinction coefficients ±5%.

^d^Fluorescence quantum yields ±10%.

^e^Fluorescence lifetimes ±10%.

^f^Not determined.

It was shown that lengthening the conjugated system of the chromophore increased the molar absorptivity of **3b** and **3bH+** and the inclusion of a thiophene ring imposed a detrimental effect on **3c** and **3cH+** molar absorptivity (tables 1 and 3). These effects are unexpected as the addition of a thiophene ring has been noted to generally increase molar absorptivity [24,25]; while increasing the length of the conjugated system has been shown to have the opposite effect [26]. Across the series of compounds, trends in fluorescence quantum yield can be seen (tables 1 and 3). The decreased fluorescence quantum yield with increased conjugation length can be correlated to the higher degree of flexibility in the structure, allowing for more rotation and vibration, though the inversion of this trend in the neutral forms is equally counterintuitive.

With the increased conjugation length, an increase in photodecomposition quantum yield also occurs (table 3). Although a longer wavelength was utilized for the excitation (405 nm), the neutral forms exhibit lower photostability than their conjugate acids. Despite this, 10% TFA/DCM solutions of the compounds were noted to degrade between experiments, though this is more likely to be a case of chemical stability in the present of an acid.

**Figure 10.**
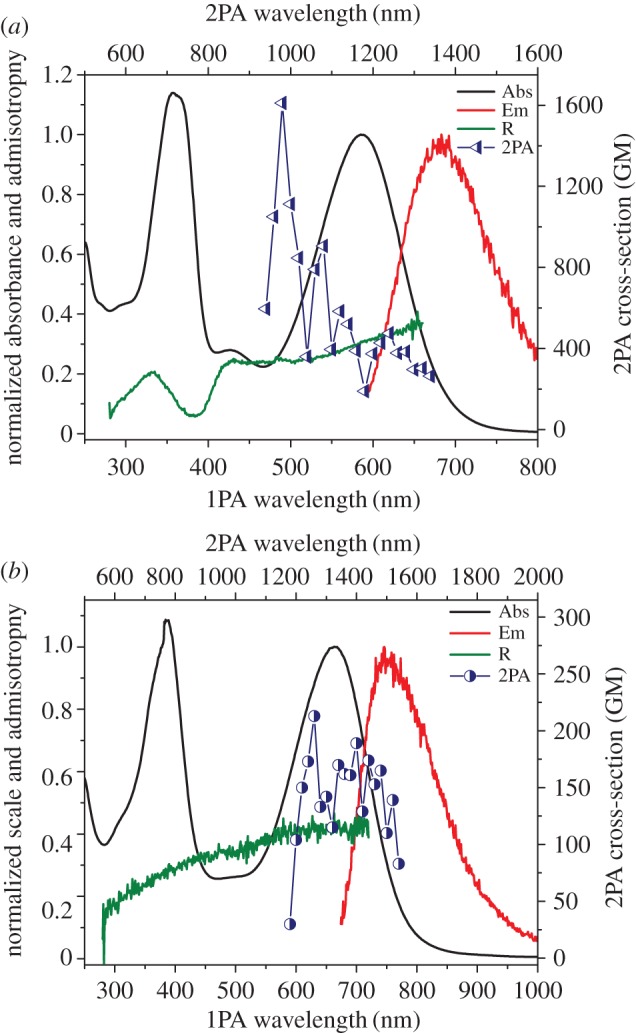
Absorption (black), emission (red), and 2PA spectra (dark blue points) for **3bH+** (*a*) and **3cH+** (*b*) in 10% TFA/DCM, and excitation anisotropy trace (dark green) in acidified silicone oil.

The excitation anisotropy trace calculated for **3bH+** presents a gradient within the region of the long wavelength absorption band, inferring that this is an overlap of more than one electronic transition (figure 10*a*). The two-photon absorption (2PA) spectrum obtained in 10% TFA/DCM of **3bH+** seems erratic, which may be explained by a variety of possible transitions. Unlike **3bH+**, the excitation anisotropy of **3cH+** plateaus within the long wavelength absorption band, in accordance with a sole transition. This plateau also suggests that the absorption and emission are collinear, given its anisotropy value of 0.4 [18]. The 2PA spectrum of **3cH+** initially shows reasonable agreement with the linear absorption spectrum, though appears erratic beyond 1300 nm (figure 10*b*).

In an effort to resolve the difference observed between the experimental results and those of the B3LYP TD-DFT calculation (figures 7 and 8), a series of additional methods were implemented by varying the proportion of Hartree–Fock (HF) calculations. *Ab initio* methods, like HF, yield results that are less erratic than semiempirical methods, though at the cost of time [27]. Hybrid functions, including B3LYP (20% HF), have been developed to bridge this gap, where the ratios can be adjusted. With respect to spectroscopic data, increasing the *ab initio* portion decreases, or blue shifts, the wavelengths of the transitions.

**Figure 11.**
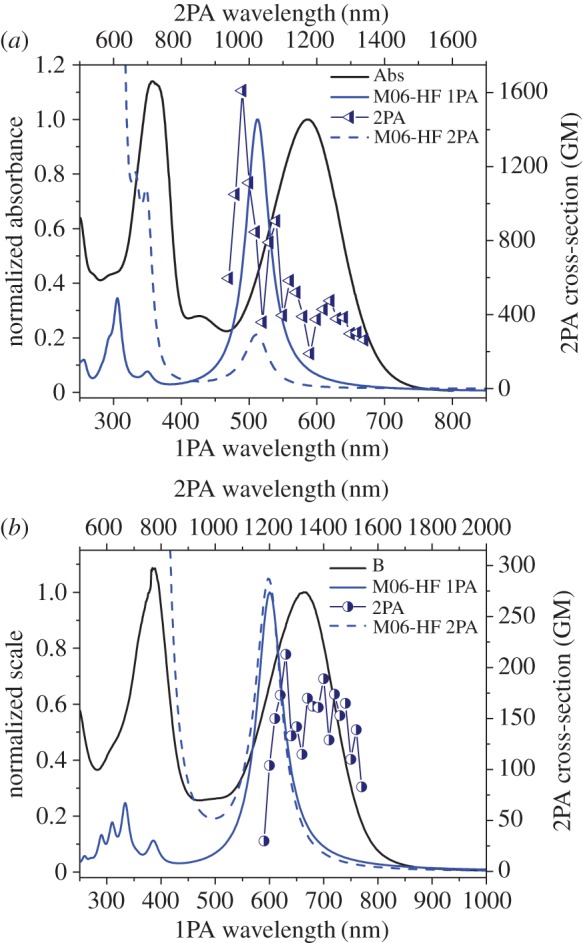
Experimental (black) and M06-HF calculated one-photon absorption (solid blue), and experimental (dark blue points) and M06-HF calculated two-photon absorption (dashed blue) for **3bH+** (*a*) and **3cH+** (*b*).

When the TD-DFT calculation was repeated for **3bH+** using the M06-HF method (100% HF), the calculated spectrum was in closer semblance to the experimental spectrum than previous results (figure 11*a*). The first transition appeared hypsochromically shifted from the experimental main absorption band, as is the shorter wavelength transition. There is greater difference in the experimental and calculated 2PA spectrum; in the region of the main linear absorption band, accounting for the wavelength shift, they were similar, though at higher energy the data diverges.

Likewise, the results obtained for **3cH+** using the same M06-HF method show a hypsochromic shift compared with the experimental data, for both the one- and two-photon absorption spectra (figure 11*b*). Here, the 2PA data are more closely matched, with results becoming erratic towards the limit of the germanium detectors (1600 nm).

The use of a method with a higher proportion of HF provided greater similarity between the calculated and experimental results for these compounds because of the presence of the large dipole moments that are more akin to charge transfer, a phenomenon that traditional DFT methods struggle to depict [28,29].

## 2. Experimental

### 2.1. General information

Reagents and solvents were purchased from commercial sources and used without further purification unless otherwise specified. TLC was performed on SiO_2_-60 F254 aluminium plates with visualization by UV light or TFA staining. Flash column chromatography was performed using a Teledyne CombiFlash Rf 200 unit. Melting points (mp.) were determined on a Fisher Scientific melting point apparatus. In total, 400 (100) MHz ^1^H (^13^C) NMR spectra were recorded on Bruker Avance III 400 spectrometer. In total, 500 (125) MHz ^1^H (^13^C) NMR were recorded on Varian VNMRS 500 spectrometer. Chemical shifts (*δ*) are given in parts per million (ppm) relative to TMS and referenced to residual protonated solvent (CDCl_3_: *δ*H 7.26 ppm, *δ*C 77.23 ppm). Abbreviations used are s (singlet), d (doublet), t (triplet), q (quartet), quin (quintet), hp (heptet), b (broad) and m (multiplet). ESI-TOF-MS spectra were recorded on Agilent 6210 TOF spectrometer. 4-(Hexyloxy)benzaldehyde **2** [30], 1-(hexyloxy)-4-vinylbenzene **4** [28], 7-bromo-9,9-dihexyl-9H-fluorene-2-carbaldehyde **6** [29] were prepared according to literature procedures.

### 2.2. Synthesis

#### 2.2.1. (*E*)-9,9-Dihexyl-7-(4-(hexyloxy)styryl)-9*H*-fluorene-2-carbaldehyde (**7**)

Pd(OAc)_2_ (0.05 mmol) was added to a solution of 1-(hexyloxy)-4-vinylbenzene **4** (1.50 mmol), 7-bromo-9,9-dihexyl-9*H*-fluorene-2-carbaldehyde **6** (1.00 mmol), K_2_CO_3_ (2.0 mmol) and Bu_4_NBr (2.00 mmol) in DMF (10 ml) under Ar. The reaction mixture was heated at 140°C for 8 h, upon which the solvent was evaporated, and the resulting crude was suspended in DCM (25 ml) and passed through a silica plug (25 g). The solvent was evaporated and the crude product was purified using flash column chromatography (hexanes : DCM, 9 : 1) to afford the title product as colourless oil that crystallized upon standing (60%). ^1^H NMR (400 MHz, CDCl_3_) *δ* 10.05 (s, 1H), 7.87 (dd, *J* = 1.4, 0.7 Hz, 1H), 7.84 (dd, *J* = 7.8, 1.4 Hz, 1H), 7.79 (dd, *J* = 7.7, 0.6 Hz, 1H), 7.74–7.71 (m, 1H), 7.20–7.04 (m, 2H), 6.94–6.88 (m, 2H), 3.98 (t, *J* = 6.6 Hz, 2H), 2.03 (ddd, *J* = 10.5, 6.4, 3.9 Hz, 4H), 1.79 (ddt, *J* = 9.0, 7.9, 6.4 Hz, 2H), 1.53–1.42 (m, 2H), 1.40–1.29 (m, 4H), 1.21–0.98 (m, 13H), 0.97–0.87 (m, 2H), 0.74 (t, *J* = 7.0 Hz, 6H), 0.61 (tq, *J* = 11.1, 7.0 Hz, 4H). ^13^C NMR (101 MHz, CDCl_3_) *δ* 192.3, 159.1, 152.7, 147.4, 138.8, 135.1, 130.6, 129.8, 128.8, 127.8, 126.6, 125.6, 123.0, 121.2, 120.6, 119.8, 114.8, 68.1, 55.2, 40.3, 31.6, 29.6, 29.3, 25.7, 23.8, 22.6, 14.1.

#### 2.2.2. (*E*)-9,9-Dihexyl-2-(4-(hexyloxy)styryl)-7-vinyl-9*H*-fluorene (**8**)

The title compound was prepared starting from intermediate **7** according to literature procedure [31]. Colourless oil (70%). ^1^H NMR (400 MHz, CDCl_3_) *δ* 7.66 (dd, *J* = 7.8, 6.1 Hz, 2H), 7.55–7.37 (m, 6H), 7.20–7.04 (m, 2H), 6.94 (d, *J* = 8.7 Hz, 2H), 6.84 (dd, *J* = 17.6, 10.9 Hz, 1H), 5.83 (d, *J* = 17.6 Hz, 1H), 5.29 (d, *J* = 10.9 Hz, 1H), 4.02 (t, *J* = 6.6 Hz, 2H), 2.10–1.93 (m, 4H), 1.83 (dt, *J* = 14.7, 6.7 Hz, 2H), 1.51 (ddt, *J* = 13.0, 9.7, 5.8 Hz, 2H), 1.39 (dq, *J* = 7.2, 3.6 Hz, 4H), 1.21–1.03 (m, 12H), 1.00–0.93 (m, 3H), 0.79 (t, *J* = 7.0 Hz, 6H), 0.74–0.64 (m, 4H). ^13^C NMR (101 MHz, CDCl_3_) *δ* 159.0, 151.7, 151.5, 141.0, 140.4, 137.6, 136.8, 136.5, 130.3, 127.8, 127.7, 127.3, 125.5, 125.4, 120.6, 120.6, 120.0, 119.8, 114.9, 113.1, 68.3, 55.1, 40.7, 31.8, 31.7, 29.9, 29.4, 25.9, 23.9, 22.8, 22.8, 14.2, 14.2.

### 2.3. General procedure for the preparation of guaiazulenes **3a-c** and **10**

A solution of *t*-BuOK (3.0 mmol) in *t*-amyl alcohol (10 ml) was heated at 105°C for 30 min. To this solution was added the appropriate aldehyde (3.0 mmol) followed by the dropwise addition of a solution of guaiazulene **1** (1.0 mmol) in *t*-amyl alchol (5 ml). The solution was heated for 3 h at 105°C, then cooled to room temperature and poured into dilute HCl (1 N). The mixture was extracted with DCM (2 × 25 ml) and the solvent was removed under reduced pressure. The resulting crude mixture was purified using Flash column chromatography using hexanes and EtOAc (0–3%).

#### 2.3.1. (*E*)-2-Bromo-5-(2-(7-isopropyl-1-methylazulen-4-yl)vinyl)thiophene (**10**)

Green needles (mp. 72–74°C, 25%). ^1^H NMR (400 MHz, CDCl_3_) *δ* 8.21 (d, *J* = 1.9 Hz, 1H), 7.80–7.68 (m, 2H), 7.51 (dd, *J* = 11.1, 2.0 Hz, 1H), 7.47–7.37 (m, 3H), 7.04 (d, *J* = 3.8 Hz, 1H), 6.94 (d, *J* = 4.0 Hz,1H), 3.13 (p, *J* = 6.9 Hz, 1H), 2.71 (s, 3H), 1.42 (d, *J* = 6.9 Hz, 6H). ^13^C NMR (101 MHz, CDCl_3_) *δ* 144.6, 140.9, 140.4, 137.1, 136.7, 136.5, 134.9, 133.3, 130.8, 129.5, 127.6, 126.2, 126.1, 119.8, 112.6, 112.0, 38.5, 29.9, 13.2. HRMS (ESI) (*m*/*z*): [M + H]^+^ calcd for C_20_H_19_BrS C_28_H_35_O 370.0391; found, 370.0408.

#### 2.3.2. (*E*)-4-(4-(Hexyloxy)styryl)-7-isopropyl-1-methylazulene (**3a**)

Green needles (mp. 55.0–58.0°C, 43%). ^1^H NMR (CDCl_3_, 400 MHz) *δ* 8.21 (s, 1H), 7.92 (d, *J* = 16.1 Hz, 1H), 7.69 (d, *J* = 3.9 Hz, 1H), 7.60 (d, *J* = 8.6 Hz, 2H), 7.57–7.49 (m, 3H), 7.38 (d, *J* = 16.1 Hz, 1H), 6.97 (d, *J* = 8.6 Hz, 2H), 4.04 (t, *J* = 6.6 Hz, 2H), 3.14 (sept, *J* = 6.9 Hz, 1H), 2.72 (s, 3H), 1.84 (dd, *J* = 8.5, 6.4 Hz, 2H), 1.56–1.33 (m, 13H), 1.00–0.93 (m, 2H). ^13^C NMR (CDCl_3_, 100 MHz) *δ* 159.7, 142.4, 139.9, 136.8, 136.6, 136.3, 135.0, 133.7, 133.1, 130.0, 128.5, 127.3, 125.9, 120.3, 115.0, 112.0, 68.3, 38.4, 31.7, 29.9, 29.4, 25.9, 24.9, 22.8, 14.2, 13.2. HRMS (ESI) (*m*/*z*): [M + H]^+^ calcd for C_28_H_35_O 387.2682; found, 387.2688.

#### 2.3.3. 9,9-Dihexyl-2-((*E*)-4-(hexyloxy)styryl)-7-((*E*)-2-(7-isopropyl-1-methylazulen-4-yl)vinyl)-9*H*-fluorene (**3b**)

Green solid (25%, 72.0–75.0°C). ^1^H NMR (CDCl_3_, 400 MHz): *δ* 8.20 (d, *J* = 1.4 Hz, 1H), 8.06 (d, *J* = 16.1 Hz, 1H), 7.73–7.61 (m, 4H), 7.59–7.44 (m, 9H), 7.19–7.04 (m, 2H), 6.95–6.87 (m, 2H), 3.99 (t, *J* = 6.6 Hz, 2H), 3.12 (p, *J* = 6.9 Hz, 1H), 2.70 (s, 3H), 2.04 (dd, *J* = 11.3, 5.7 Hz, 4H), 1.87–1.73 (m, 2H), 1.48 (dd, *J* = 10.5, 4.8 Hz, 2H), 1.40 (d, *J* = 6.9 Hz, 6H), 1.38–1.31 (m, 4H), 1.16–1.03 (m, 11H), 0.95–0.90 (m, 4H), 0.81–0.65 (m, 10H). ^13^C NMR (CDCl_3_, 100 MHz): *δ* 159.2, 152.1, 152.0, 140.3, 137.0, 136.4, 135.2, 135.0, 130.5, 128.9, 128.1, 128.0, 127.4, 126.2, 125.8, 121.7, 120.9, 120.6, 120.4, 120.3, 115.1, 77.6, 68.5, 55.4, 40.9, 38.7, 32.0, 31.9, 30.1, 29.6, 26.1, 25.1, 24.2, 23.0, 14.4, 14.4, 13.4. Calcd for C_55_H_68_O [M + H]^+^ = 745.5343; found [M + H]^+^ = 745.5189.

#### 2.3.4. 2-((*E*)-2-(9,9-Dihexyl-7-((*E*)-4-(hexyloxy)styryl)-9*H*-fluoren-2-yl)vinyl)-5-((*E*)-2-(7-isopropyl-1-methylazulen-4-yl)vinyl)thiophene (**3c**)

Green solid (39%, 154.0–156.0°C). ^1^H NMR (CDCl_3_, 400 MHz): *δ*.18 (d, *J* = 1.8 Hz, 1H), 7.82 (d, *J* = 15.8 Hz, 1H), 7.73–7.60 (m, 3H), 7.53–7.41 (m, 10H), 7.27 (dd, *J* = 16.0, 0.7 Hz, 1H), 7.19–7.00 (m, 5H), 6.90 (d, *J* = 8.7 Hz, 2H), 3.98 (t, *J* = 6.6 Hz, 2H), 3.10 (p, *J* = 6.9 Hz, 1H), 2.72–2.64 (s, 3H), 2.07–1.93 (m, 4H), 1.85–1.75 (m, 2H), 1.52–1.43 (m, 2H), 1.37 (m, 10H), 1.19–0.99 (m, 11H), 0.95–0.88 (m, 4H), 0.80–0.73 (t, *J* = 8.0 Hz, 6H), 0.73–0.63 (m, 4H). Calcd for C_55_H_68_O [M + H]^+^ = 852.5304; found [M + H]^+^ = 852.5373.

### 2.4. Photophysical characterization

Linear photophysical properties were measured in solution (approx. 10^−6 ^M) in 10 mm quartz cuvettes using spectroscopic grade solvent. An Agilent 8453 was used to collect absorption spectra, while an Edinburgh Instruments FLS 980 for steady-state luminescence emission, excitation anisotropy and fluorescence lifetimes. These measurements used a red-sensitive photomultiplier tube (PMT), and a liquid-nitrogen-cooled Hamamatsu R5509–72; all measurements were corrected for detector response.

Fluorescence quantum yields were calculated using a relative method, with 9,10-DPA (*Φ_f_* = 0.95) as a reference [18]. Anisotropy measurements were performed in a viscous solvent, namely silicone oil, to hamper the rotational relaxation of the molecules. Fluorescence lifetimes were measured using a 470 nm laser for **3aH**, a 510 nm laser for **3bH** and a 670 nm laser for **3cH**.

Photodecomposition quantum yields, *Φ*_Ph_*,* were measured by irradiating into the main absorption band of solutions with a LOCTITE 97034 UV-lamp (λ_ex_ = 366 nm, *I*_0_(λ)* *≈* *13 mW cm^−2^), a green diode laser (λ_ex_ = 532 nm, *I*_0_(λ)* *≈* *98 mW cm^−2^), or a red diode laser (λ_ex_ = 650 nm, *I*_0_(λ)* *≈* *62 mW cm^−2^). Spectra were recorded at incremental time intervals, and the data were utilized in equation (2.1), where *D*(λ, 0) and *D*(λ,*t*_ir_) are the initial and final optical density of the solution, ε(λ) is the extinction coefficient (dm^3 ^mol^−1 ^cm^−1^), *t* is irradiation time (s) and λ is excitation wavelength (cm), *N*_A_ is Avogadro's number, *t*_ir_ is total irradiation time, *I*_0_(λ) is the spectral distribution of the excitation irradiance [32].

2.1 $$\Phi_{\mathrm{Ph}}=\frac{[D(\lambda ,0)-D(\lambda ,t_{\mathrm{ir}})]N_{A}}{10^{3}\varepsilon (\lambda )\int_{\lambda }\int_{0}^{t_{\mathrm{ir}}}I_{0}(\lambda )[1-10^{D(\lambda ,t)}]\;d\lambda \;dt}$$

Two-photon absorption spectra were collected through an open aperture z-scan set-up (previously detailed [33]) using solutions (10^−2 ^M) in a 1 mm cuvette translated through the focal point of the output beam of an optical parametric amplifier (OPA) pumped by a 1 kHz, approximately 100 fs Coherent, Inc. Legend Elite, that was seeded by a Coherent, Inc. Mira Ti:sapphire laser [34].

### 2.5. Quantum chemical calculations

Taking the output from the GaussView5 program, structures were optimized to the B3LYP/D95* level with the Gaussian09 software package [35]. TD-DFT calculations looking at the first 15 transitions utilized these optimized conformations, and, initially, the B3LYP method. Further calculations used M05, M06, M06-2X and M06-HF methods. The alkoxy and alkyl chains in the *para*-position of phenyl and 9-position of fluorene were shortened to methoxy and methyl groups, respectively, due to the minimal impact these groups have on the results of the calculations [36]. The calculated 2PA spectrum was determined from the permanent and state-to-state transition dipoles, which were obtained using *a posteriori* Tamm–Dancoff approximation (ATDA) in a locally modified version of Gaussian09 [37].

## 3. Conclusion

Through the use of quantum chemical calculations, a library of guaiazulene derivatives based on an initial synthesized compound led to the discovery of molecules with high linear absorption dipoles. Measurements on two synthesized structures from the series illustrated issues in the calculations performed. As a result of the numerous terms that factor into nonlinear absorption, the larger dipole did not correspond to the great 2PA.

Despite the discrepancies between experimental and computational results, the use of quantum chemical calculations to consider the properties of designed, yet unsynthesized, compounds is promising. Conducting *in silico* analysis and prediction, combined with deriving materials from renewable resources, is a noteworthy course of action for chemists in this increasingly ecologically and sustainability-conscious world.

## Supplementary Material

supplementary information of computational and NMR data

## Data accessibility

Computational data and molecular orbital illustrations along with ^1^H and ^13^C NMR spectra are provided in the electronic supplementary material available from the publisher.
