# Supplementary material for: Computer aided chemical design: using quantum chemical calculations to predict properties of a series of halochromic guaiazulene derivatives
Source: R Soc Open Sci. 2016 Nov 23;3(11):160373. doi: 10.1098/rsos.160373 (PMC5180112; doi:10.1098/rsos.160373)
Supplement: supplementary information of computational and NMR data [file rsos160373supp1.pdf]

Computer Aided Chemical Design: Using Quantum Chemical Calculations to Predict Properties of a Series of Halochromic Guaiazulene Derivatives

Adam W. Woodward,[1] Ebrahim H. Ghazvini Zadeh,[1] Mykhailo V. Bondar,[2] and Kevin D. Belfield*[3,4]

[1]*Department of Chemistry, University of Central Florida, Orlando, FL 32816-2366, USA*

[2]*Institute of Physics NASU, Prospect Nauki, 46, Kiev-28, 03028, Ukraine*

[3]*College of Science and Liberal Arts, New Jersey Institute of Technology, University Heights, Newark, NJ 07102, USA*

[4]*School of Chemistry and Chemical Engineering, Shaanxi Normal University, Xi'an, 710062, PRC*

* To whom correspondence should be addressed e-mail: belfield@njit.edu

TD-DFT data S2 – S7

Frontier molecular orbitals S8 – S10

^{1}H and ^{13}C NMR spectra S11 – S19

Table S1: Calculated properties for the first 15 transitions for **3a** as determined by TD-DFT[a]

Transition no.	Energy /eV	Wavelength /nm	Oscillator strength	Leading configuration	
1	1.97	629	0.03	H -> L	0.96
2	3.08	403	0.43	H-1 -> L	0.81
				H -> L+1	0.15
3	3.31	375	0.13	H-2 -> L	0.31
				H-1 -> L	0.14
				H -> L+1	0.51
4	3.75	331	0.02	H-2 -> L	0.25
				H -> L+1	0.03
				H -> L+2	0.58
				H -> L+3	0.11
5	3.88	320	0.30	H-1 -> L+1	0.90
6	4.02	309	0.03	H -> L+2	0.25
				H -> L+3	0.65
7	4.23	293	0.22	H-2 -> L	0.22
				H-2 -> L+1	0.13
				H-1 -> H+2	0.14
				H -> L+1	0.15
				H -> L+3	0.17
8	4.32	287	0.01	H-3 -> L	0.48
				H-1 -> L+2	0.25
				H-1 -> L+3	0.17
9	4.54	273	0.02	H-4 -> L	0.36
				H-2 -> L	0.42
10	4.67	265	0.04	H-3 -> L	0.38
				H-1 -> L+2	0.32
11	4.90	253	0.60	H-2 -> L	0.10
				H-1 -> L+2	0.12
				H-1 -> L+3	0.58
12	5.04	246	0.26	H-4 -> L	0.42
				H-2 -> L+1	0.22
				H-2 -> L+2	0.13
13	5.22	238	0.12	H-2 -> L+2	0.72
14	5.36	231	0.07	H-2 -> L+3	0.74
15	5.49	226	0.02	H-5 -> L	0.56
				H-4 -> L+1	0.19

[a] H = HOMO, L = LUMO

Table S2: Calculated properties for the first 15 transitions for **3aH** as determined by TD-DFT[a]

Transition no.	Energy /eV	Wavelength /nm	Oscillator strength	Leading configuration	
1	2.38	522	0.27	H -> L	0.36
				H -> L+1	0.63
2	2.52	493	0.71	H -> L	0.64
				H -> L+1	0.36
3	3.27	379	0.00	H-1 -> L	0.91
4	3.43	361	0.07	H-2 -> L	0.77
5	3.65	340	0.07	H-2 -> L+1	0.63
				H-1 -> L+1	0.21
6	3.82	324	0.01	H-2 -> L+1	0.20
				H-1 -> L+1	0.77
7	3.89	318	0.16	H-3 -> L	0.82
				H -> L+2	0.11
8	4.22	294	0.05	H-3 -> L	0.84
9	4.47	277	0.22	H -> L+2	0.73
10	4.74	262	0.01	H-4 -> L	0.58
11	4.78	259	0.09	H -> L+3	0.24
				H-4 -> L	0.21
12	4.99	249	0.00	H -> L+3	0.54
13	5.06	245	0.16	H-5 -> L	0.96
				H-6 -> L	0.27
				H-4 -> L+1	0.60
14	5.24	237	0.00	H-9 -> L	0.12
				H-7 -> L	0.80
15	5.32	233	0.00	H-9 -> L	0.57
				H-8 -> L	0.18

[a] H = HOMO, L = LUMO

Table S3: Calculated properties for the first 15 transitions for **3b** as determined by TD-DFT[a]

Transition no.	Energy /eV	Wavelength /nm	Oscillator strength	Leading configuration	
1	1.9263	643.65	0.041	H -> L	0.90
2	2.5963	477.55	1.3718	H-1 -> L	0.95
3	2.9044	426.88	0.0082	H -> L+1	0.73
4	3.1808	389.79	0.5176	H-2 -> L	0.50
				H-1 -> L+1	0.29
5	3.2689	379.28	0.5495	H-1 -> L+1	0.51
				H -> L+1	0.10
				H -> L+2	0.26
6	3.4402	360.4	0.0301	H-3 -> L	0.31
				H-2 -> L	0.24
				H-1 -> L+2	0.10
				H -> L+2	0.21
7	3.6608	338.68	0.0034	H-1 -> L+2	0.80
8	3.7297	332.42	0.0514	H-3 -> L	0.30
				H-2 -> L+1	0.36
				H -> L+2	0.12
9	3.8537	321.73	0.0094	H-2 -> L+1	0.31
				H-1 -> L+3	0.12
				H -> L+4	0.38
10	3.8633	320.93	0.0077	H-6 -> L	0.10
				H-1 -> L+3	0.27
				H-1 -> L+4	0.11
				H -> L+3	0.34
11	3.9751	311.91	0.0005	H-1 -> L+3	0.24
				H -> L+3	0.51
				H -> L+4	0.13
12	4.0233	308.16	0.0787	H-5 -> L	0.17
				H-4 -> L	0.30
				H-1 -> L+4	0.13
13	4.0811	303.8	0.0507	H-4 -> L	0.34
				H-1 -> L+4	0.12
				H -> L+4	0.12
14	4.1255	300.53	0.0618	H-5 -> L	0.18
				H-2 -> L+2	0.47
15	4.2128	294.31	0.0526	H-5 -> L	0.10
				H-1 -> L+4	0.15
				H-1 -> L+5	0.30

[a] H = HOMO, L = LUMO

Table S4: Calculated properties for the first 15 transitions for **3b** as determined by TD-DFT[a]

Transition no.	Energy /eV	Wavelength /nm	Oscillator strength	Leading configuration	
1	1.23	1009	0.86	H -> L	0.99
2	1.48	839	0.08	H -> L+1	0.97
3	2.18	569	0.81	H-1 -> L	0.91
4	2.51	494	0.08	H-1 -> L+1	0.92
5	2.57	482	0.07	H-2 -> L	0.95
6	2.71	458	0.27	H-4 -> L	0.14
				H-3 -> L	0.30
				H -> L+2	0.50
7	2.74	453	0.08	H-3 -> L	0.69
				H -> L+2	0.18
8	3.01	411	0.04	H-4 -> L	0.69
				H -> L+2	0.17
9	3.09	402	0.04	H-5 -> L	0.91
10	3.16	392	0.01	H-2 -> L+1	0.96
11	3.34	371	0.03	H-4 -> L+1	0.62
				H-3 -> L+1	0.23
12	3.38	366	0.01	H-4 -> L+1	0.19
				H-3 -> L+1	0.77
13	3.47	358	0.41	H -> L+3	0.84
14	3.54	350	0.12	H-6 -> L	0.76
15	3.67	338	0.05	H -> L+4	0.76

[a] H = HOMO, L = LUMO

Table S5: Calculated properties for the first 15 transitions for **3c** as determined by TD-DFT[a]

Transition no.	Energy /eV	Wavelength /nm	Oscillator strength	Leading configuration	
1	1.81	686	0.08	H-1 -> L	0.19
				H -> L	0.71
2	2.28	543	2.21	H-1 -> L	0.76
				H -> L	0.23
3	2.67	464	0.16	H-2 -> L	0.17
				H -> L+1	0.60
4	2.78	446	0.52	H-2 -> L	0.61
				H-1 -> L+1	0.29
5	2.95	421	0.28	H-2 -> L	0.17
				H-1 -> L+1	0.54
				H -> L+1	0.18
6	3.10	400	0.02	H-3 -> L	0.30
				H -> L+2	0.39
7	3.29	376	0.11	H-3 -> L	0.15
				H-2 -> L+1	0.55
				H -> L+2	0.16
8	3.35	370	0.01	H-1 -> L+2	0.72
9	3.43	362	0.00	H-3 -> L	0.11
				H-2 -> L+1	0.22
				H-1 -> L+3	0.26
				H -> L+2	0.22
10	3.48	356	0.03	H-4 -> L	0.10
				H-1 -> L+3	0.16
				H -> L+3	0.52
11	3.68	336	0.01	H-4 -> L	0.64
				H-2 -> L+2	0.11
12	3.77	329	0.25	H-3 -> L	0.16
				H-2 -> L+1	0.10
				H-1 -> L+2	0.18
				H-1 -> L+3	0.18
13	3.78	328	0.01	H-6 -> L	0.12
				H-1 -> L+4	0.36
				H -> L+4	0.27
14	3.85	322	0.16	H-3 -> L+1	0.18
				H-2 -> L+2	0.52
				H-1 -> L+3	0.10
15	3.96	313	0.02	H-5 -> L	0.16

Transition no.	Energy /eV	Wavelength /nm	Oscillator strength	Leading configuration	
				H-3 -> L+1	0.19
				H-2 -> L+3	0.29

[a] H = HOMO, L = LUMO

Table S6: Calculated properties for the first 15 transitions for **3cH** as determined by TD-DFT[a]

Transition no.	Energy /eV	Wavelength /nm	Oscillator strength	Leading configuration	
1	0.97	1273	0.89	H -> L	1.01
2	1.41	877	0.02	H -> L+1	0.98
3	1.78	698	1.49	H-1 -> L	0.90
4	2.13	582	0.09	H-1 -> L+1	0.92
5	2.15	578	0.30	H-2 -> L	0.28
				H -> L+2	0.65
6	2.42	511	0.09	H-3 -> L	0.27
				H-2 -> L	0.53
				H -> L+2	0.14
7	2.49	498	0.12	H-3 -> L	0.68
				H-2 -> L	0.13
8	2.55	486	0.02	H-4 -> L	0.95
9	2.83	437	0.01	H-5 -> L	0.85
10	2.86	433	0.03	H-2 -> L+1	0.83
11	2.91	426	0.31	H-6 -> L	0.38
				H-1 -> L+2	0.47
12	2.95	420	0.16	H-1 -> L+3	0.83
13	3.16	392	0.00	H-3 -> L+1	0.95
14	3.21	387	0.06	H-6 -> L	0.32
				H-1 -> L+2	0.35
				H-1 -> L+4	0.10
15	3.30	376	0.00	H-4 -> L+1	0.99

[a] H = HOMO, L = LUMO

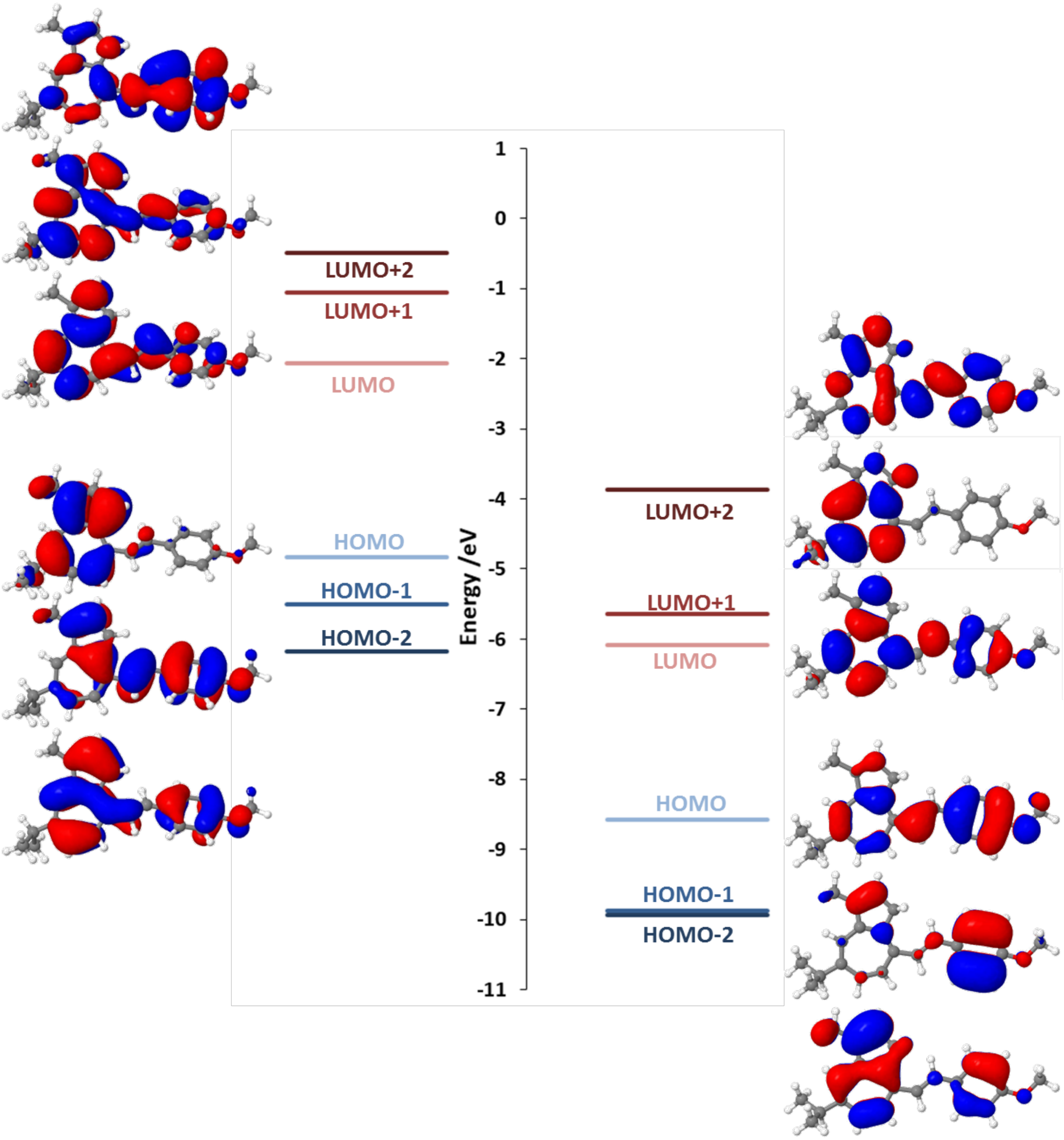

Figure S1: Frontier molecular orbitals of **3a** and **3aH+**

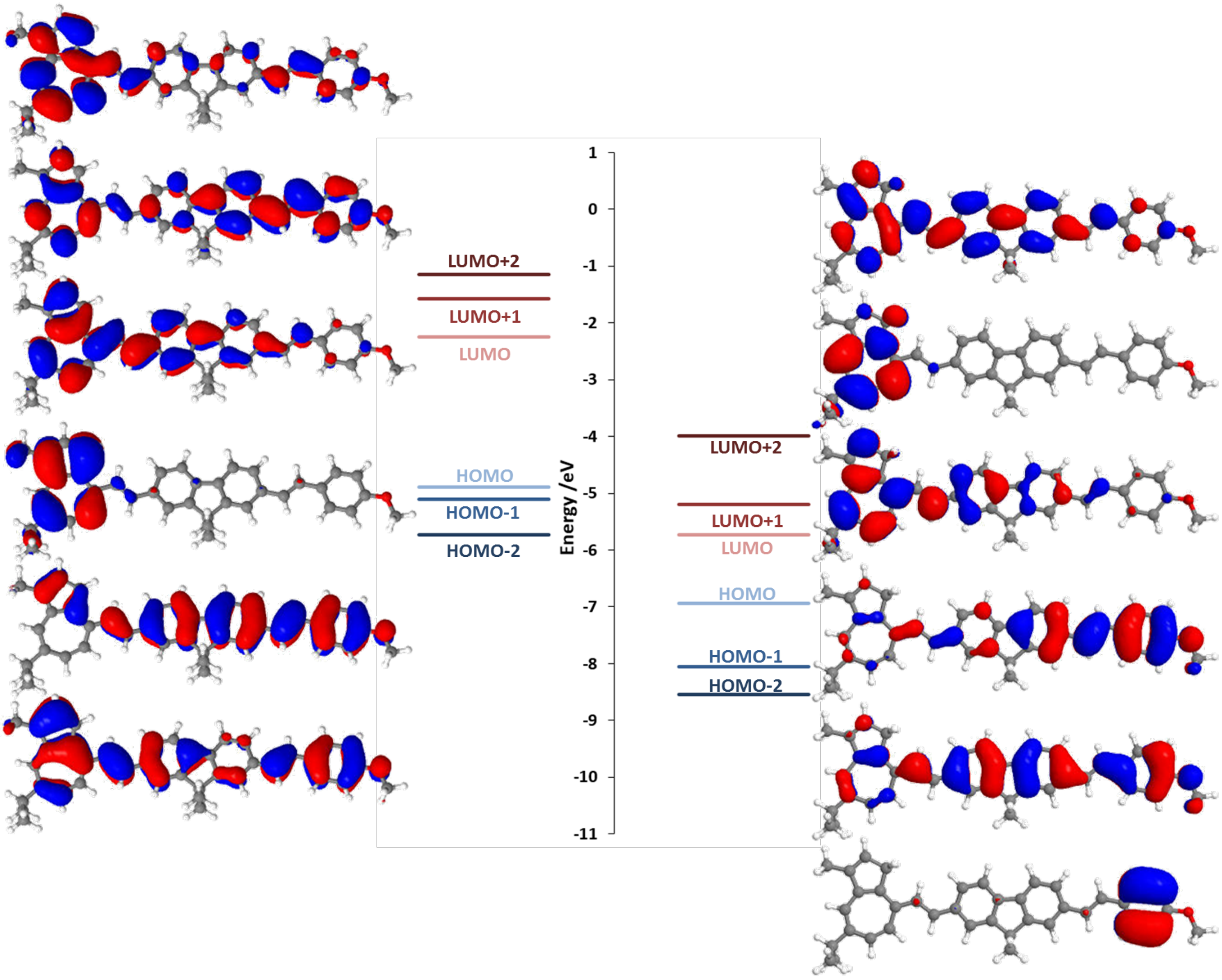

Figure S2: Frontier molecular orbitals of **3b** and **3bH+**

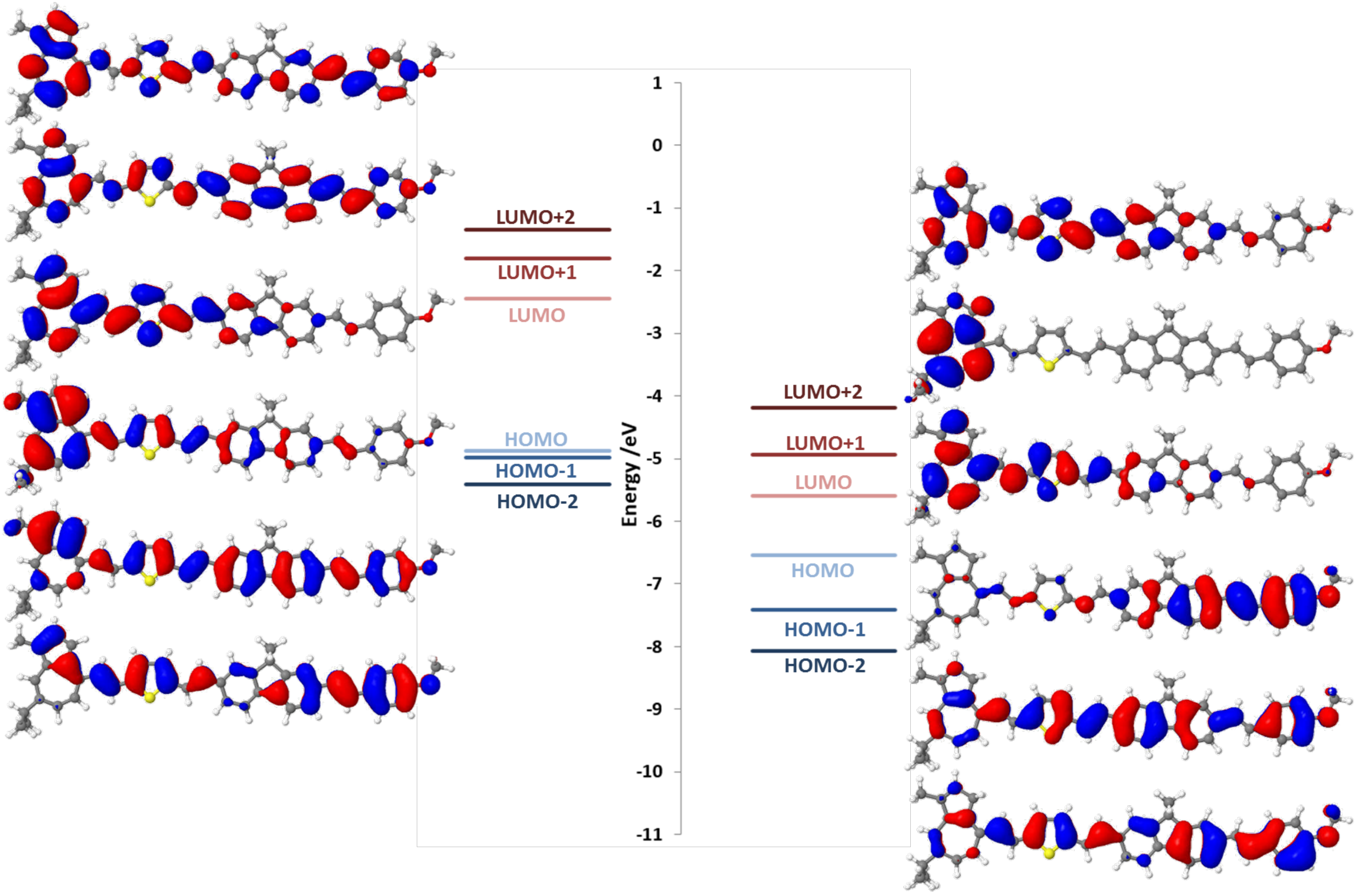

Figure S3: Frontier molecular orbitals of **3c** and **3cH+**

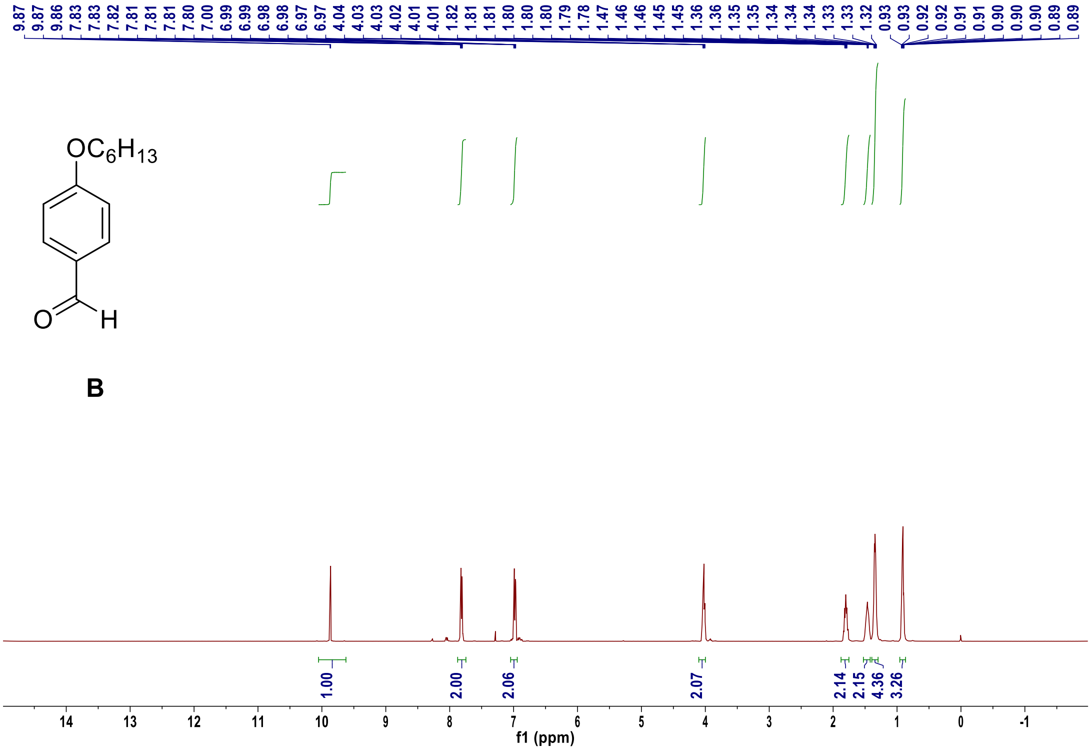

Figure S4: ^{1}H NMR spectrum for intermediate **B**

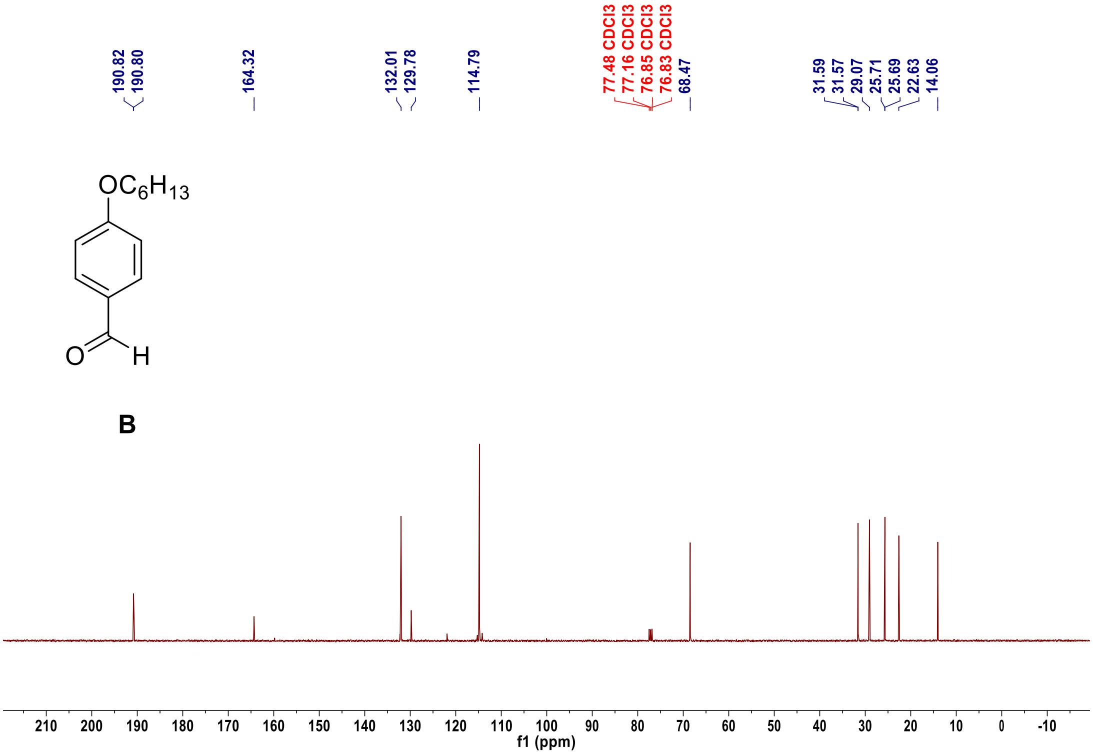

Figure S5: ^{13}C NMR spectrum for intermediate **B**

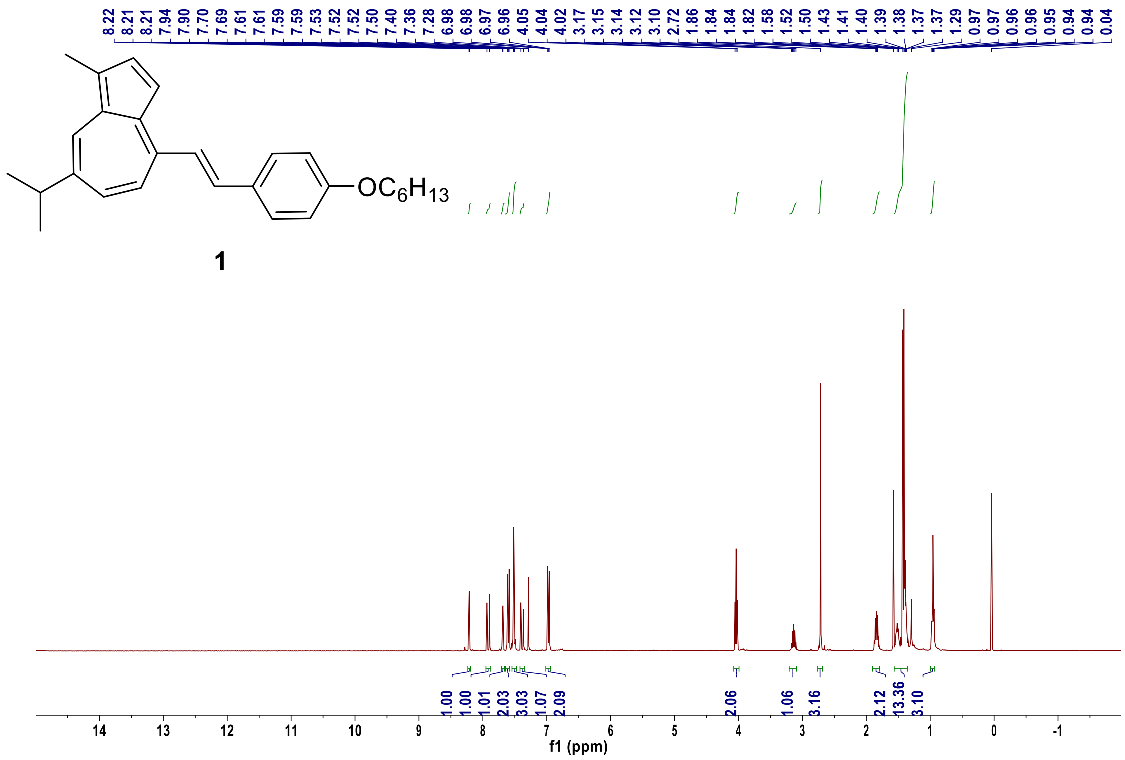

Figure S6: ^{1}H NMR spectrum for **3a**

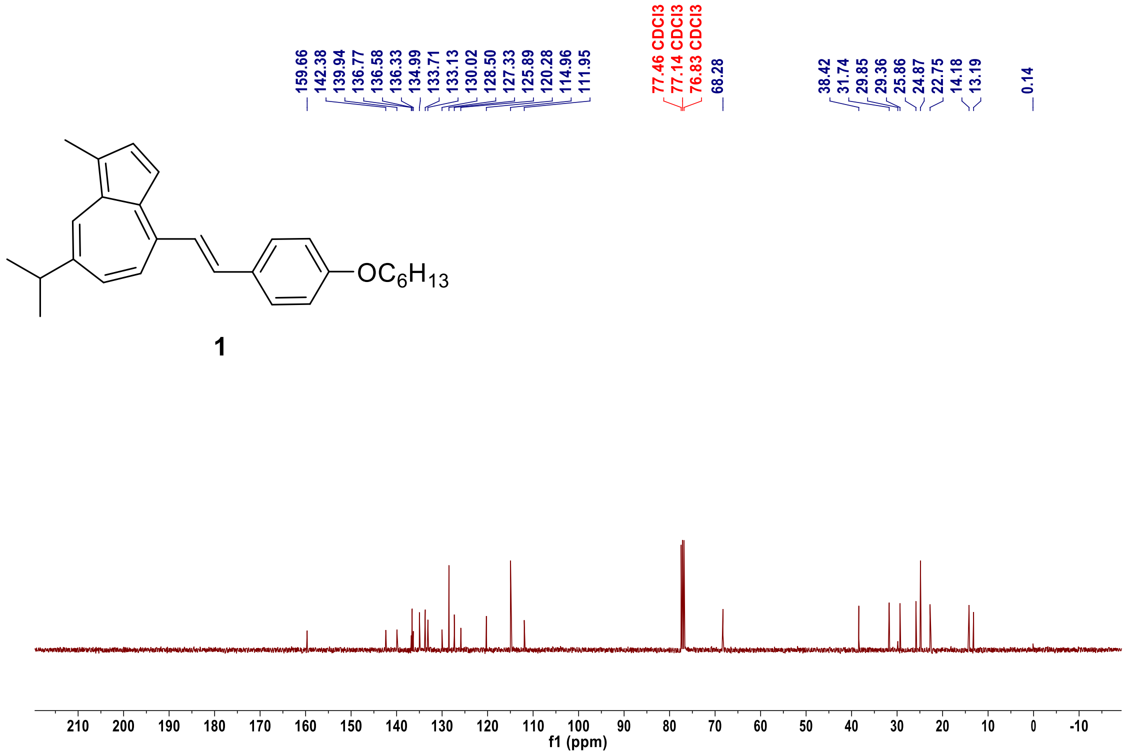

Figure S7: ^{13}C NMR spectrum for **3a**

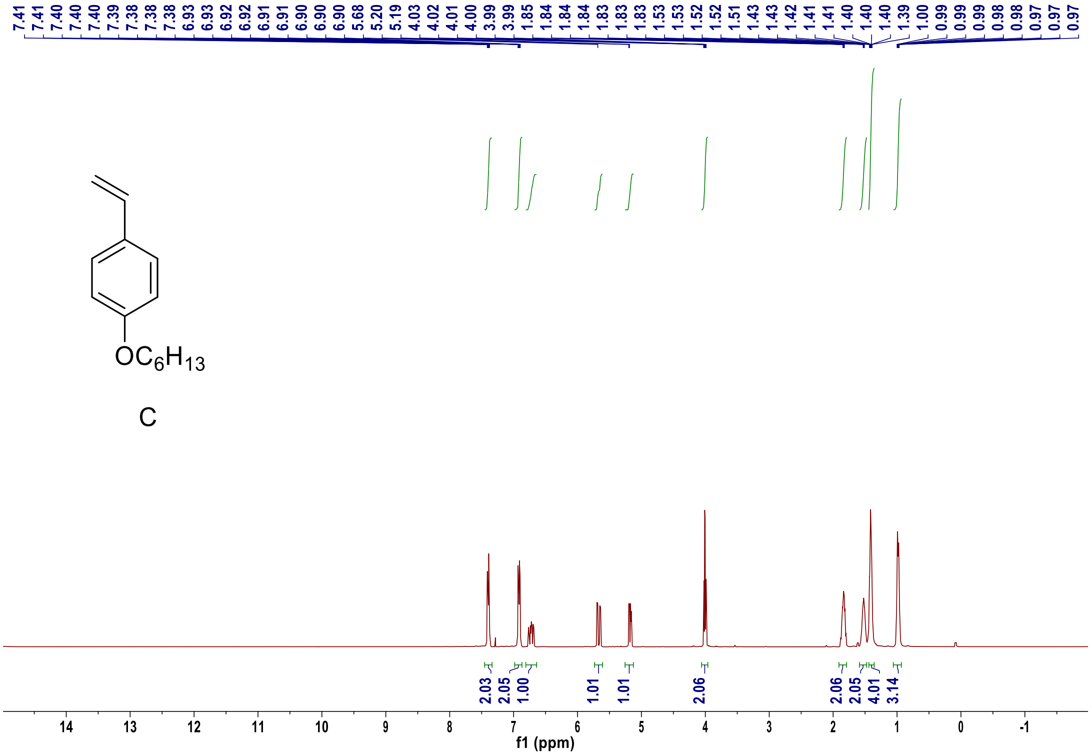

Figure S8: ^{1}H NMR spectrum for intermediate **C**

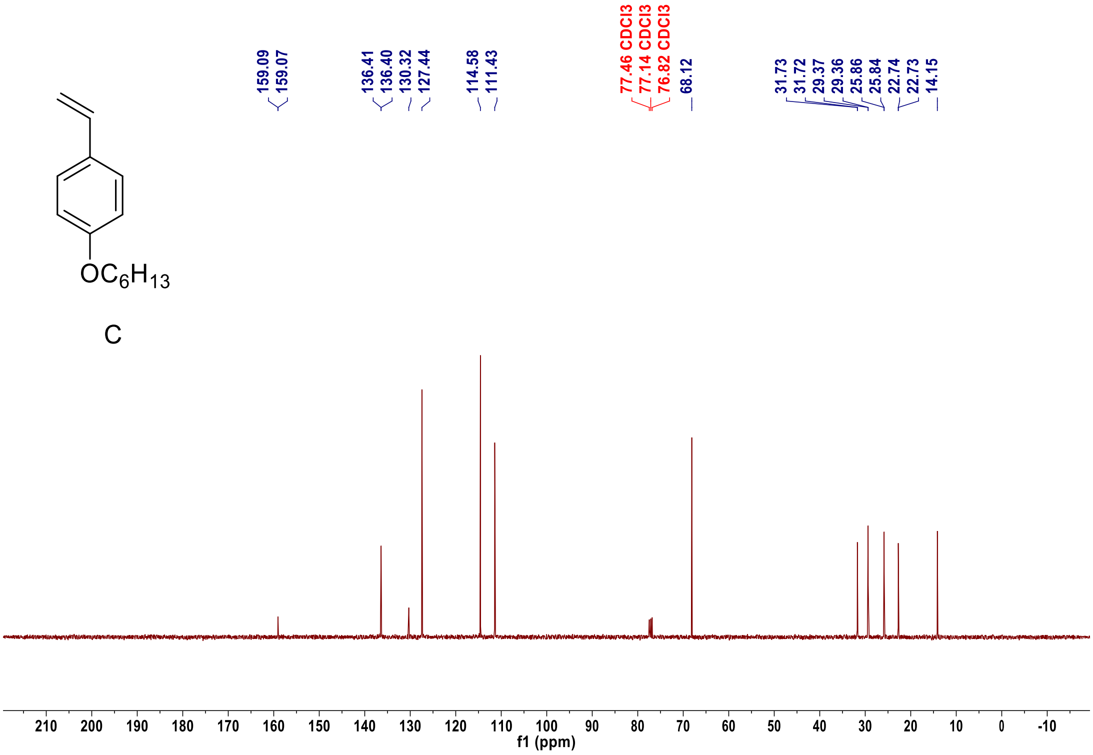

Figure S9: ^{13}C NMR spectrum for intermediate **C**

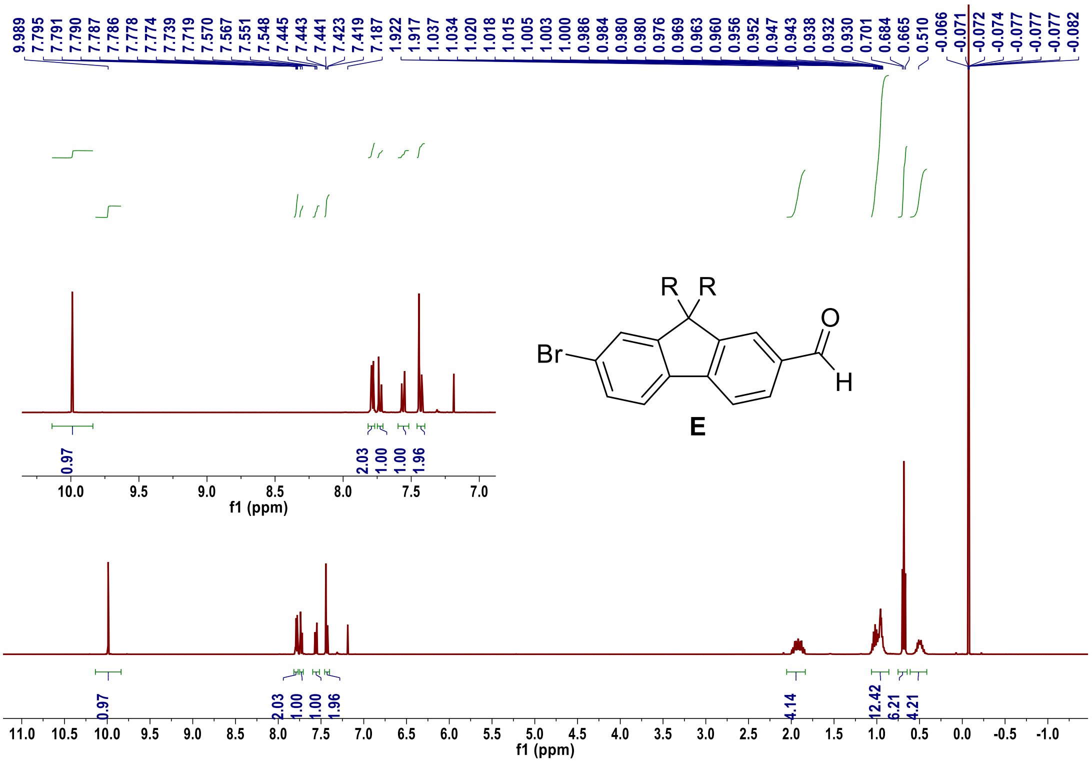

Figure S10: ^{1}H NMR spectrum for intermediate **E**

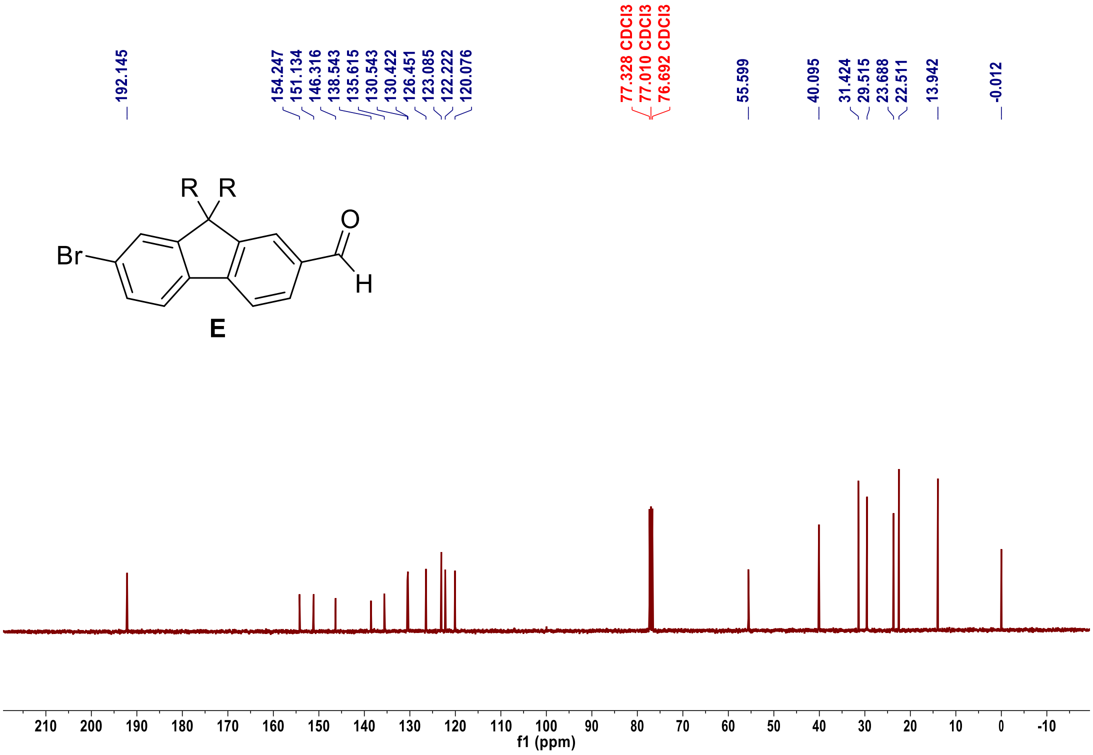

Figure S11: ^{13}C NMR spectrum for intermediate **E**

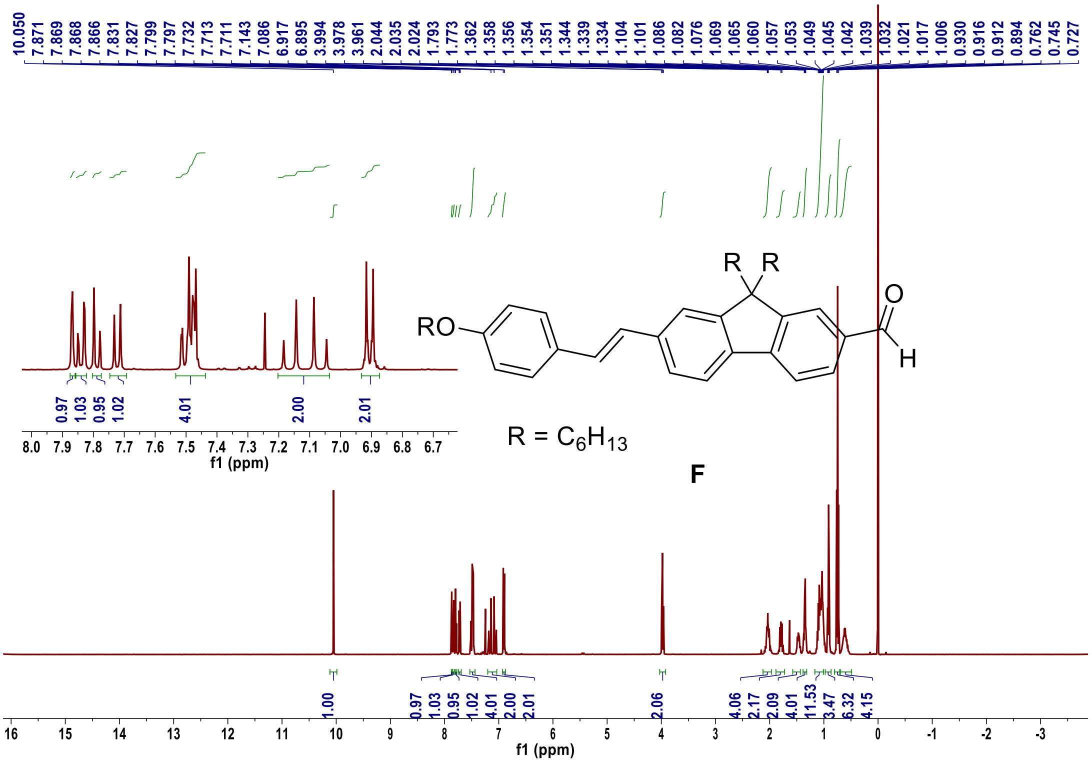

Figure S12: ¹H NMR spectrum for intermediate **F**

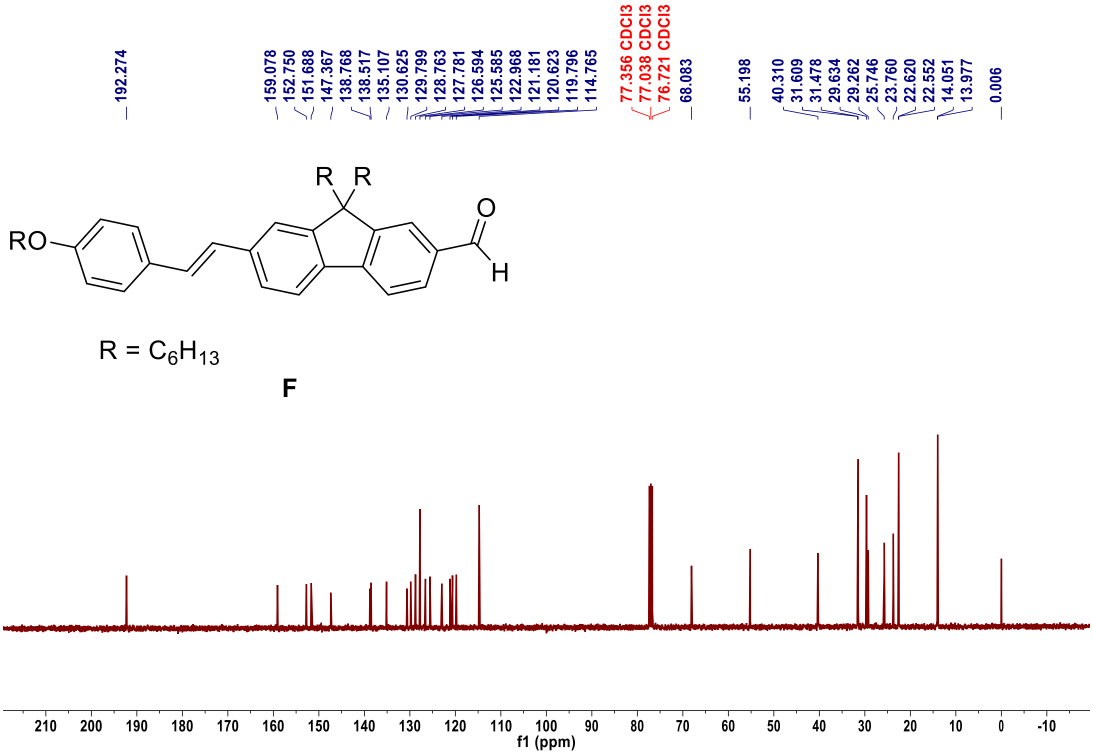

Figure S13: ¹³C NMR spectrum for intermediate F

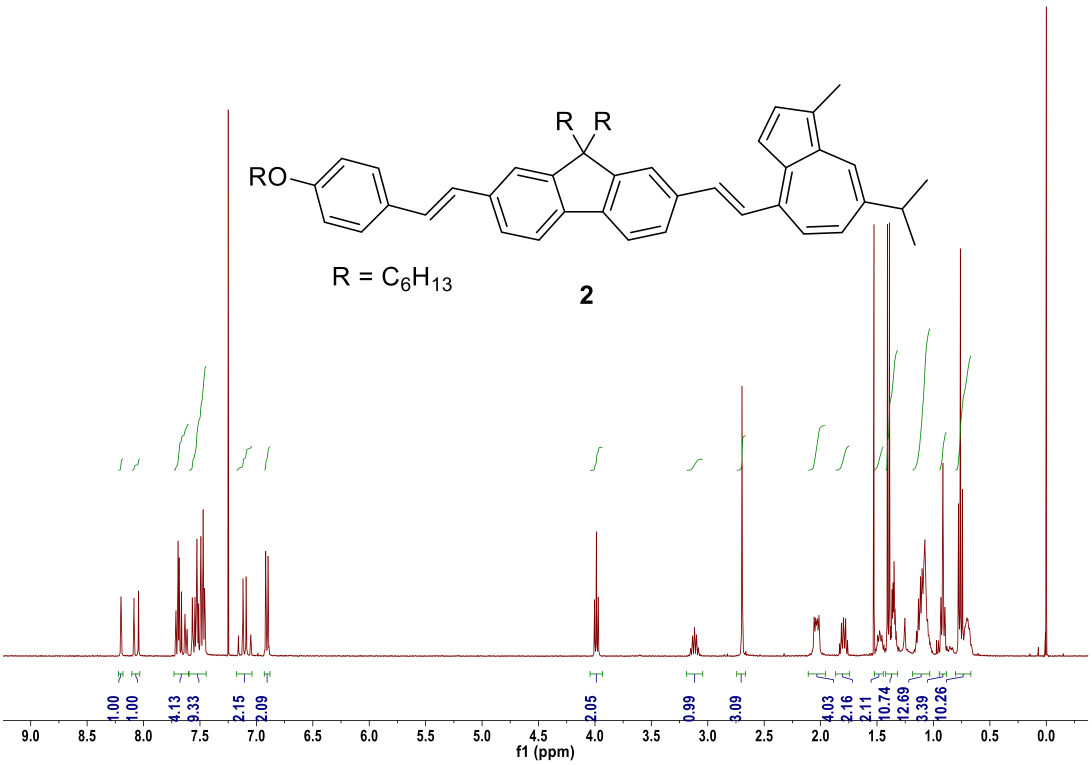

Figure S14: ^{1}H NMR spectrum for **3b**

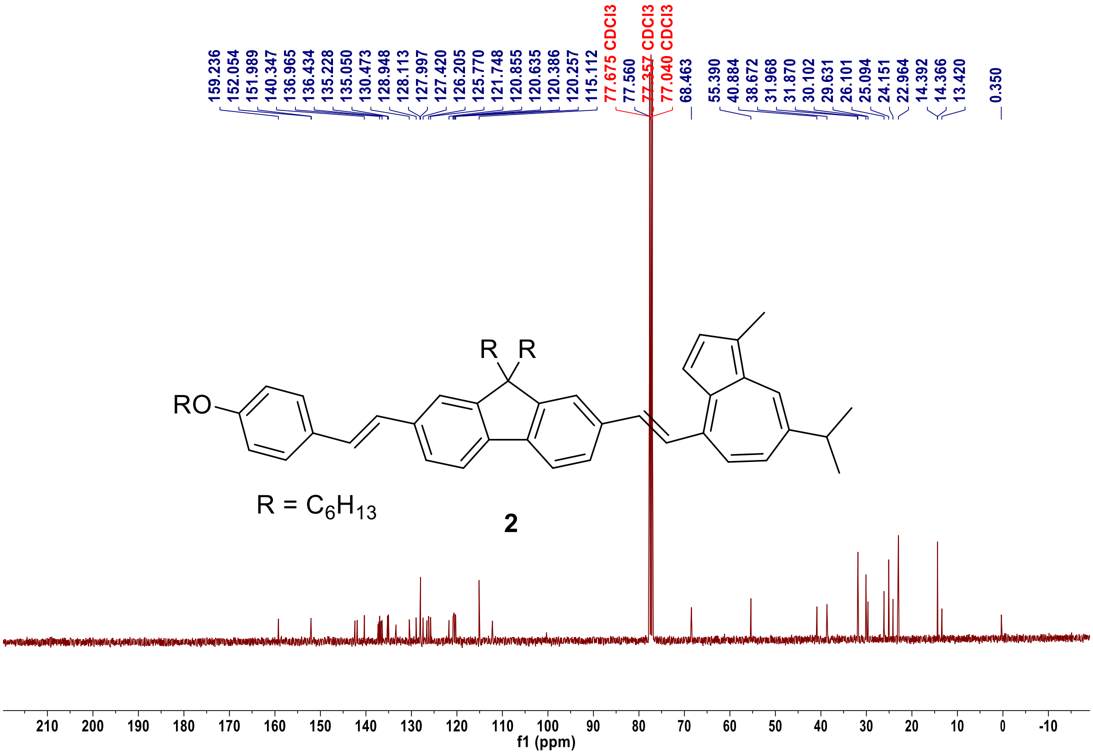

Figure S15: ^{13}C NMR spectrum for **3b**

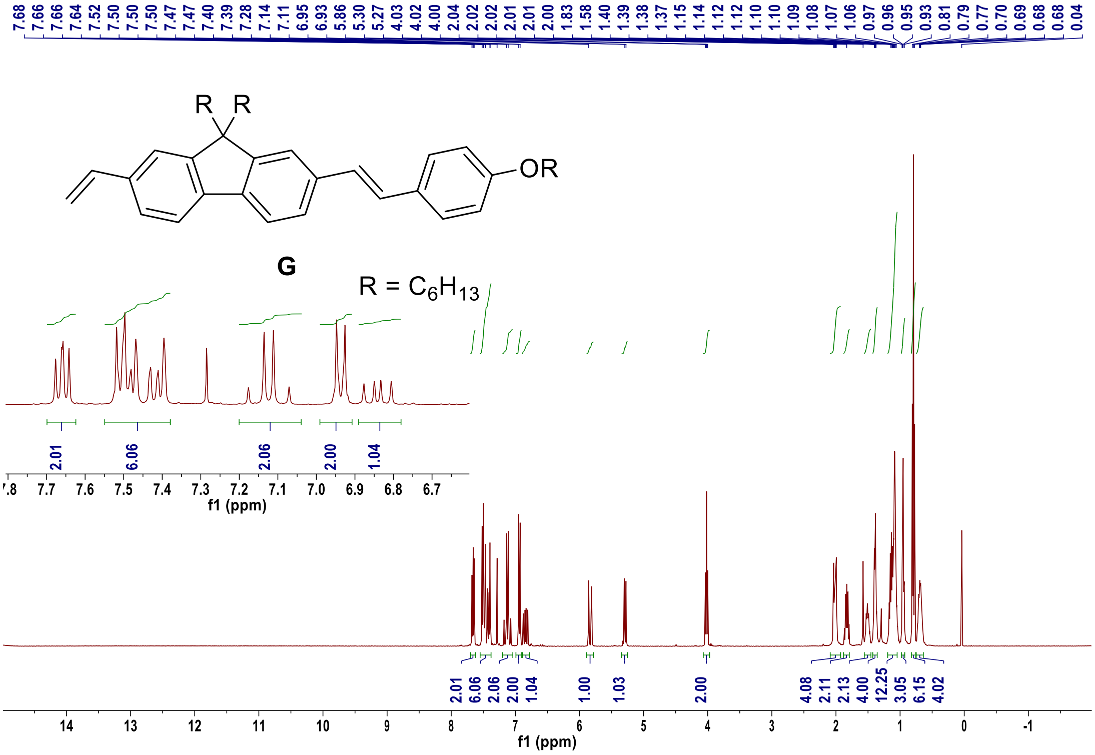

Figure S16: ^{1}H NMR spectrum for intermediate **G**

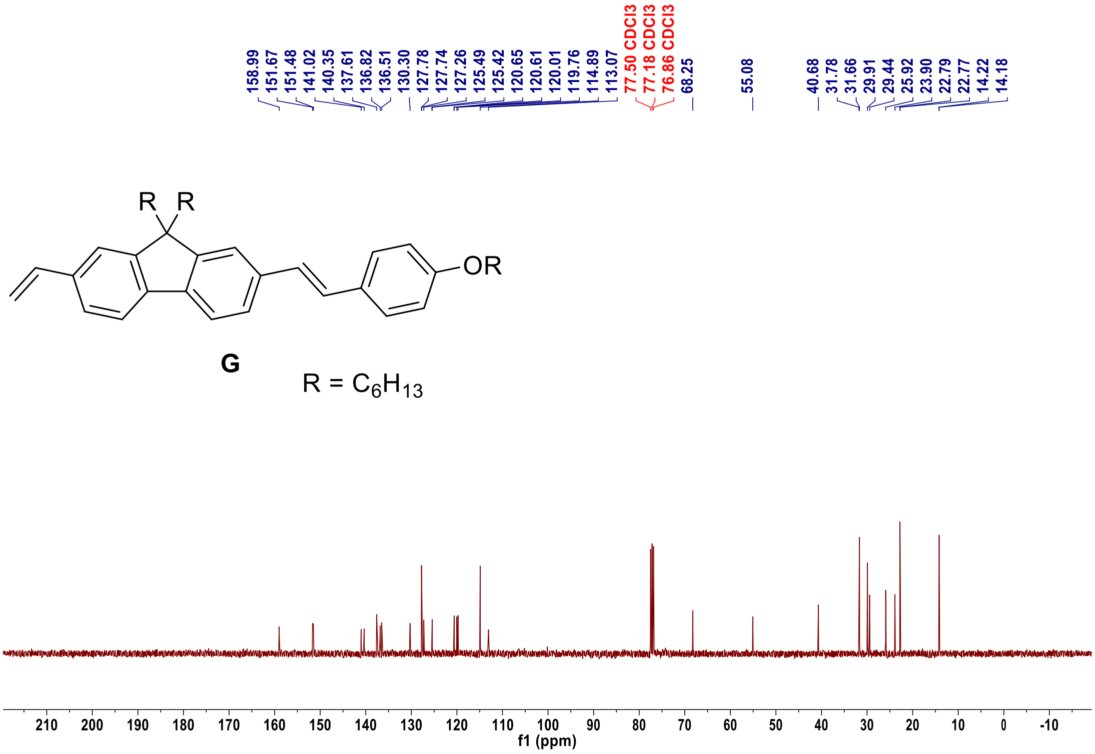

Figure S17: ^{13}C NMR spectrum for intermediate **G**

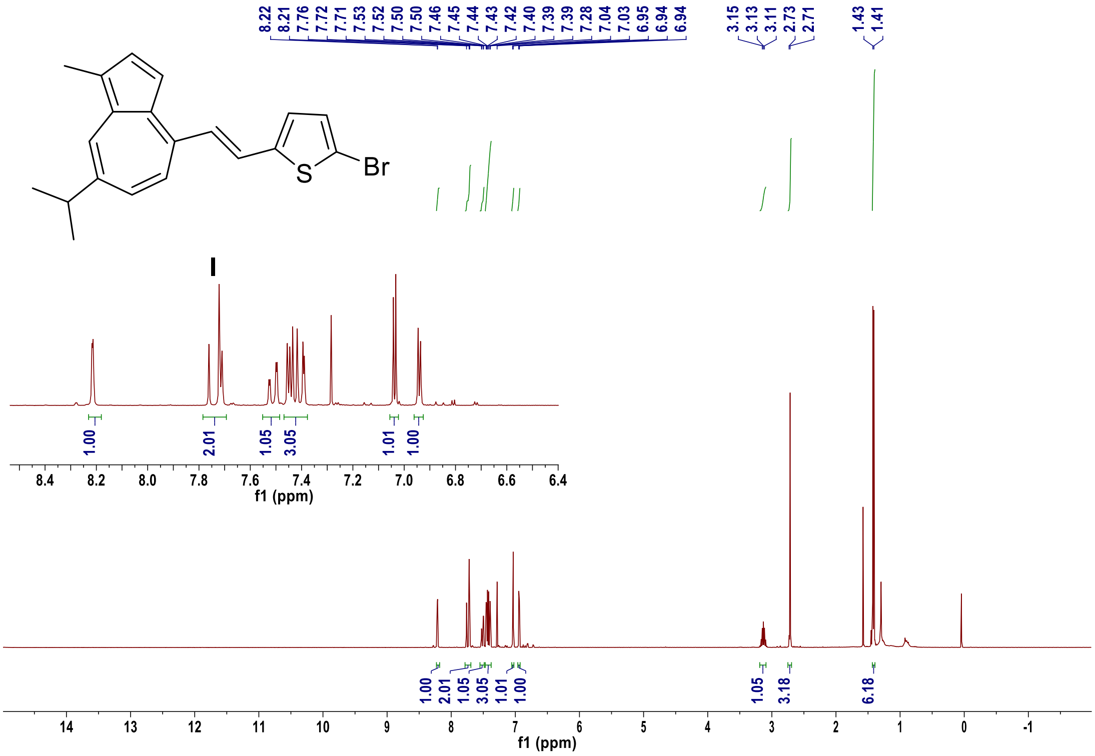

Figure S18: ^{1}H NMR spectrum for intermediate **I**

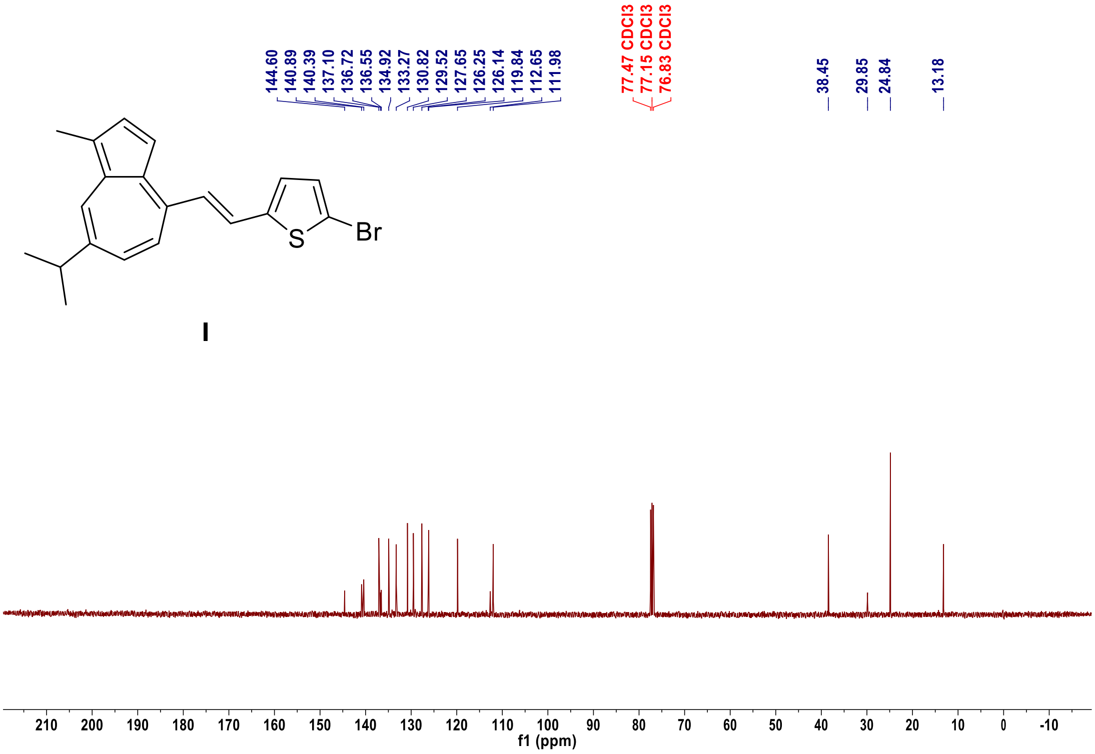

Figure S19: ^{13}C NMR spectrum for intermediate **I**

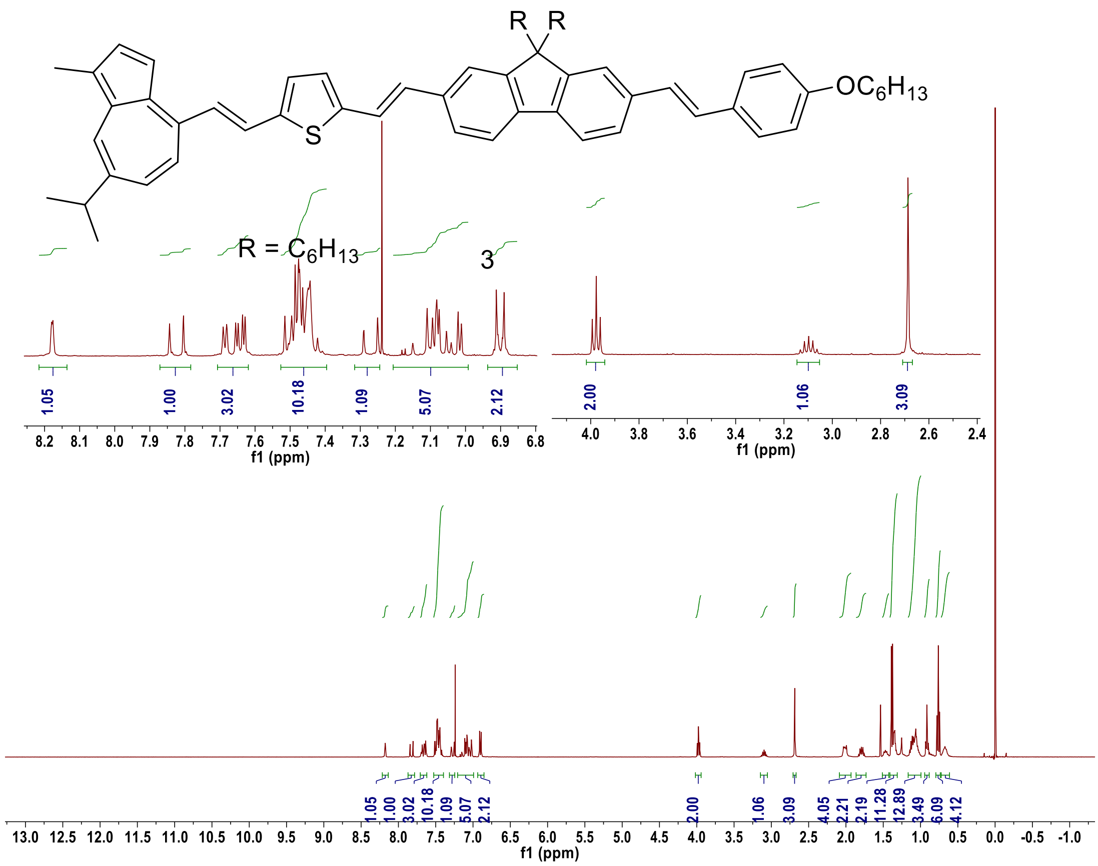

Figure S20: ^{1}H NMR spectrum for **3c**

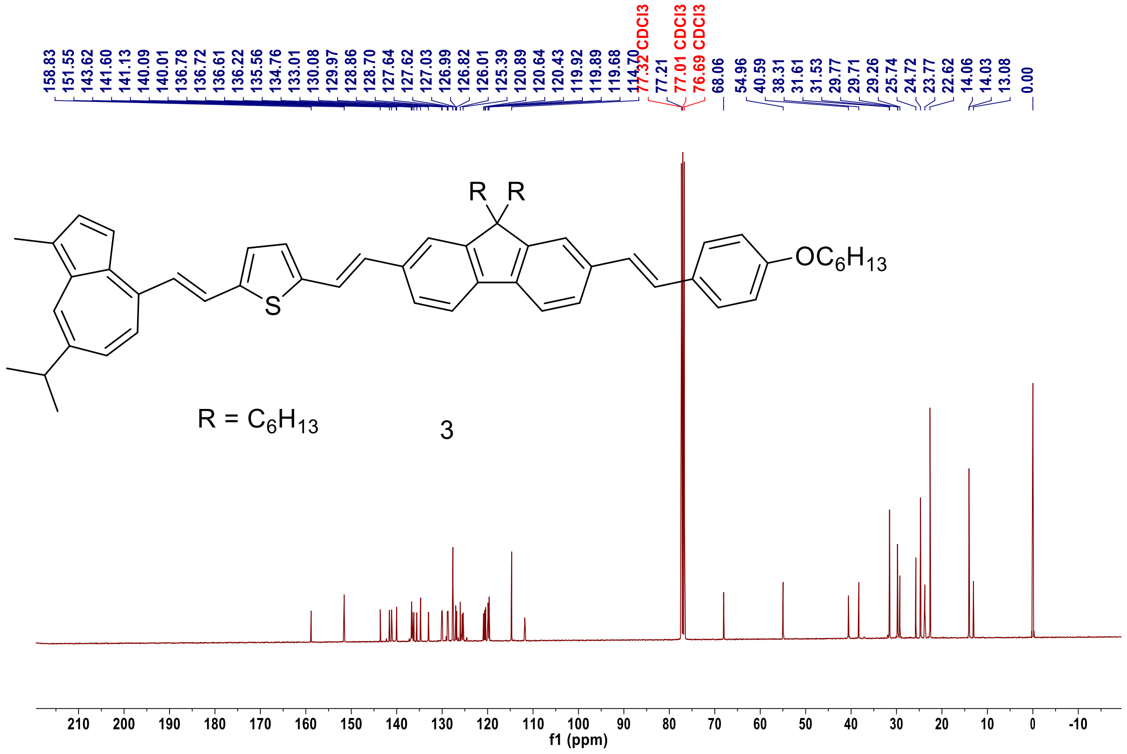

Figure S21: ^{13}C NMR spectrum for **3c**
